# Supplementary material for: Quantitative Trait Locus Mapping of Marsh Spot Disease Resistance in Cranberry Common Bean (Phaseolus vulgaris L.)
Source: Int J Mol Sci. 2022 Jul 11;23(14):7639. doi: 10.3390/ijms23147639 (PMC9324509; doi:10.3390/ijms23147639)
Supplement: Supplementary file 1 [file ijms-23-07639-s001.zip › ijms-1758465-supplementary/Figure_S2.pdf]

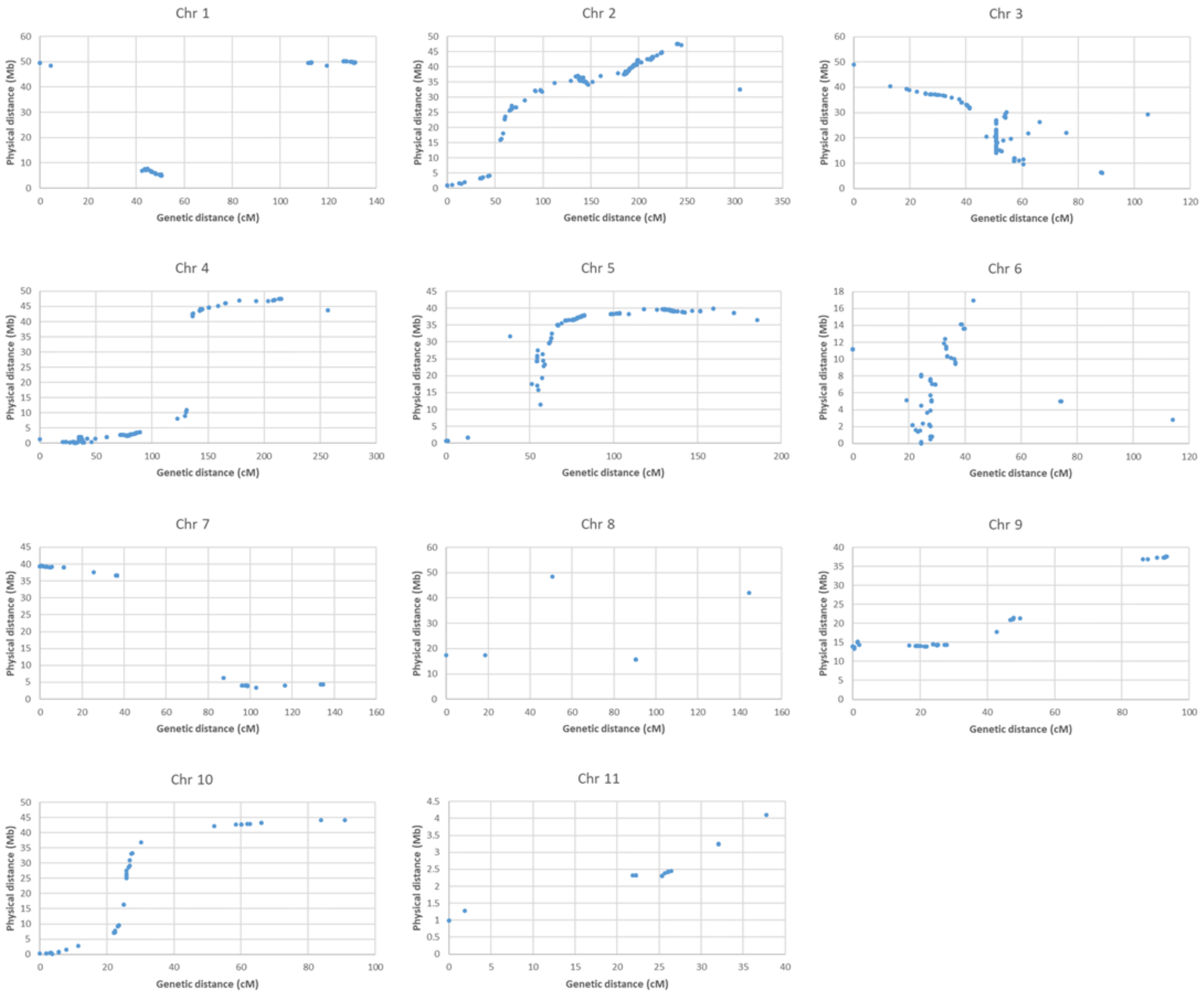

Figure S2. Relationship between genetic distance (cM) and physical distance(Mb) in 11 chromosomes. Some markers from scaffolds that have not been assigned to chromosomes and from different chromosomes were excluded.
