# Supplementary material for: Quantitative Trait Locus Mapping of Marsh Spot Disease Resistance in Cranberry Common Bean (Phaseolus vulgaris L.)
Source: Int J Mol Sci. 2022 Jul 11;23(14):7639. doi: 10.3390/ijms23147639 (PMC9324509; doi:10.3390/ijms23147639)
Supplement: Supplementary file 1 [file ijms-23-07639-s001.zip › ijms-1758465-supplementary/Figure_S3.pdf]

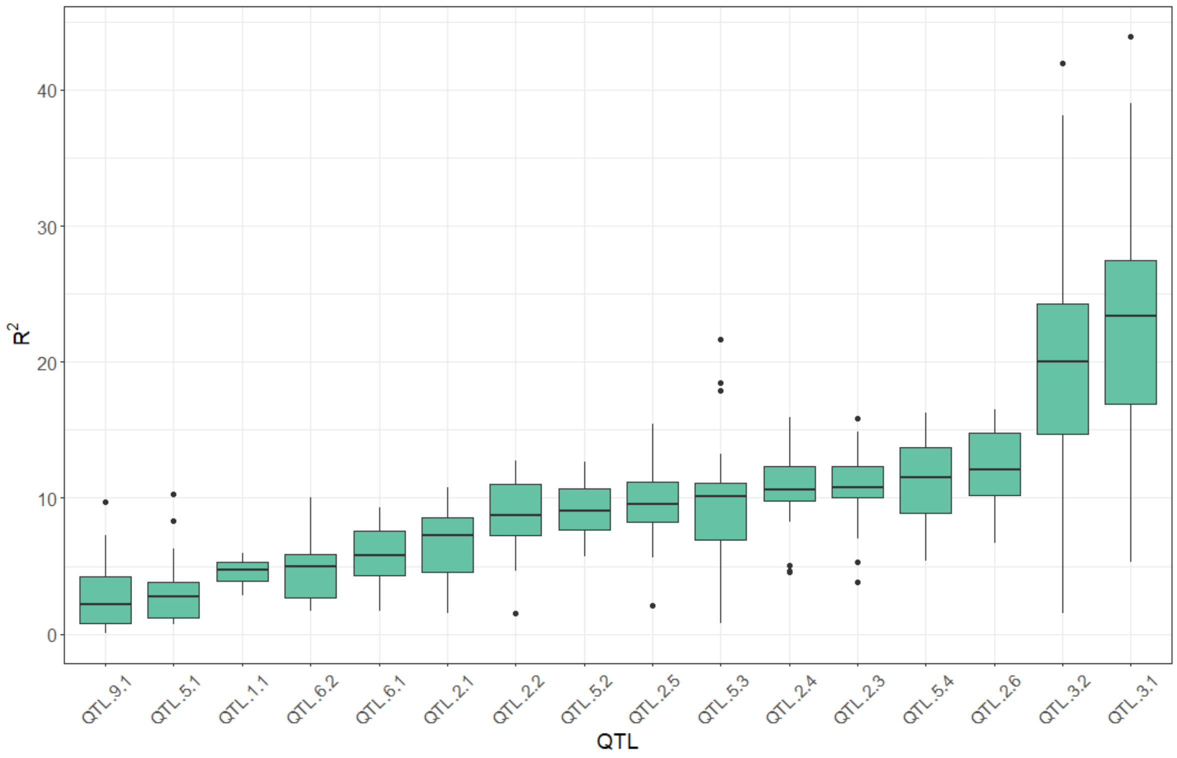

Figure S3. Boxplots of R^2 of quantitative trait loci (QTL) in 18 phenotypic data sets. Only R^2 values from the datasets that had significant correlation with QTL were used
