# Supplementary material for: Quantitative Trait Locus Mapping of Marsh Spot Disease Resistance in Cranberry Common Bean (Phaseolus vulgaris L.)
Source: Int J Mol Sci. 2022 Jul 11;23(14):7639. doi: 10.3390/ijms23147639 (PMC9324509; doi:10.3390/ijms23147639)
Supplement: Supplementary file 1 [file ijms-23-07639-s001.zip › ijms-1758465-supplementary/Figure_S4.pdf]

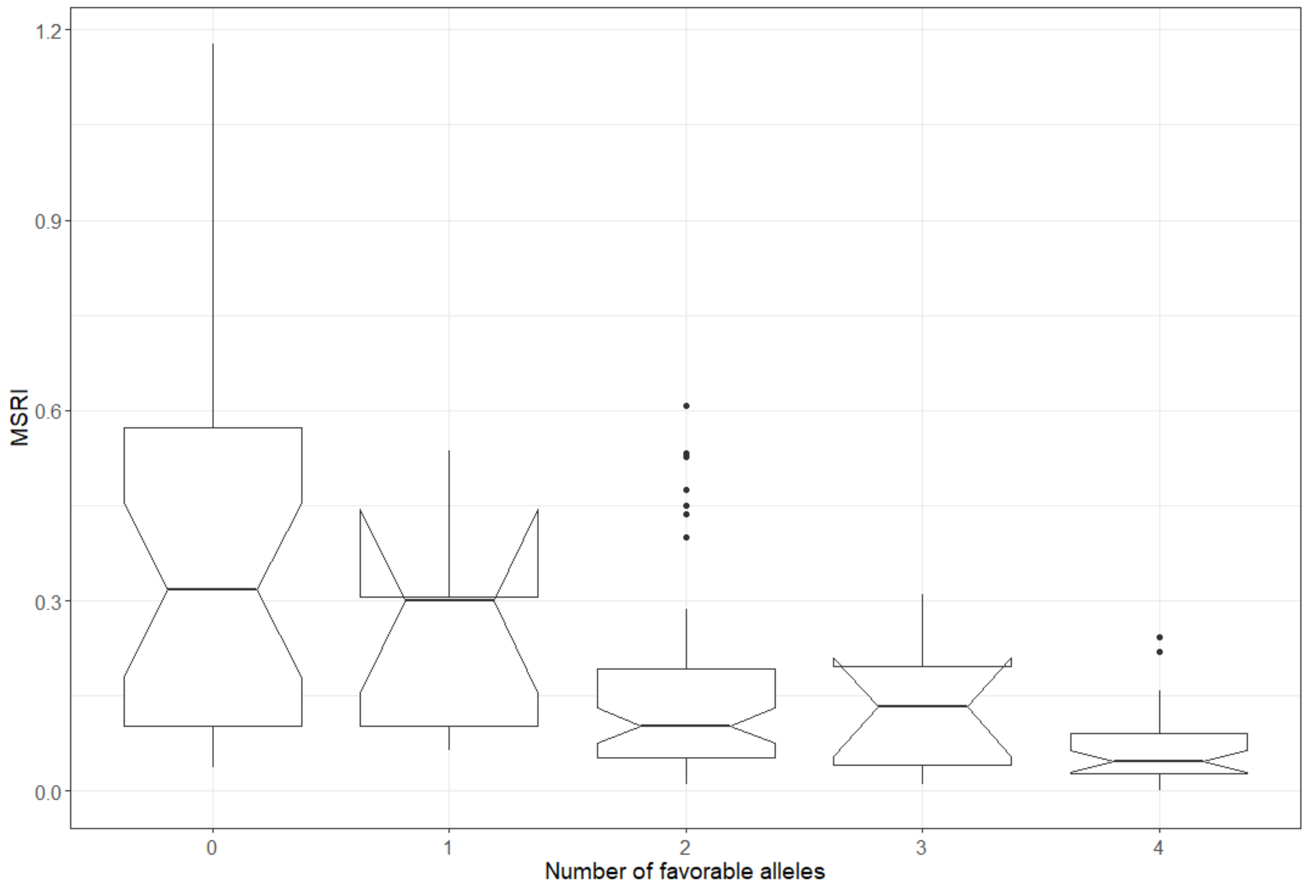

Figure S4. Relationship between the number of favorable alleles for three pairs of epistatic quantitative trait loci (QTL) and marsh spot resistance index (MSRI) of the 138 recombinant inbred lines (RILs).
