# Supplementary material for: Quantitative Trait Locus Mapping of Marsh Spot Disease Resistance in Cranberry Common Bean (Phaseolus vulgaris L.)
Source: Int J Mol Sci. 2022 Jul 11;23(14):7639. doi: 10.3390/ijms23147639 (PMC9324509; doi:10.3390/ijms23147639)
Supplement: Supplementary file 1 [file ijms-23-07639-s001.zip › ijms-1758465-supplementary/Table_S1.pdf]

Table S1. Statistics of genotyping by sequencing reads generated from the 138 recombinant inbred lines (RILs) and two parents of the cranberry common bean

Item	RILs (138)				Cran09	Messina
	Mean	Min	Max	Std		
Total reads	13,064,398	420,790	42,884,536	7,433,013	117,779,218	105,462,778
Total length (Mb)	1,959.66	63.12	6,432.68	1,114.95	17,666.88	15,819.42
Genome coverage depth	3.65	0.12	11.97	2.08	32.89	29.45
Mapped reads	10,308,751	336,808	32,483,693	5,836,050	92,079,813	84,434,975
Mapped reads %	78.56	66.54	82.7	2.15	77.87	79.75
Mapped length (Mb)	1,546.31	50.52	4,872.55	875.42	13,811.97	12,665.25
Mapped genome coverage depth	2.88	0.09	9.07	1.63	25.71	23.58
