# Supplementary material for: Quantitative Trait Locus Mapping of Marsh Spot Disease Resistance in Cranberry Common Bean (Phaseolus vulgaris L.)
Source: Int J Mol Sci. 2022 Jul 11;23(14):7639. doi: 10.3390/ijms23147639 (PMC9324509; doi:10.3390/ijms23147639)
Supplement: Supplementary file 1 [file ijms-23-07639-s001.zip › ijms-1758465-supplementary/Table_S2.pdf]

Table S2. Statistics of single nucleotide polymorphism (SNP) identification from the 138 recombinant inbred lines (RILs) and two parents

Chromosome	Total SNPs	SNPs with MAF >= 0.05, call rate ≥ 20% and polymorphic between two parents
1	3,892	174
2	6,261	377
3	4,390	162
4	5,251	203
5	5,061	318
6	2,857	100
7	3,880	95
8	6,542	105
9	3,042	111
10	3,811	93
11	7,689	150
Scaffolds	1,944	170
Total	54,620	2,058

MAF: minor allele frequency
