# Supplementary material for: Quantitative Trait Locus Mapping of Marsh Spot Disease Resistance in Cranberry Common Bean (Phaseolus vulgaris L.)
Source: Int J Mol Sci. 2022 Jul 11;23(14):7639. doi: 10.3390/ijms23147639 (PMC9324509; doi:10.3390/ijms23147639)
Supplement: Supplementary file 1 [file ijms-23-07639-s001.zip › ijms-1758465-supplementary/Table_S3.pdf]

Table S3. Linkage map of the cranberry common bean population.

Marker	Linkage group/Chromosome	Genetic distance (cM)
Chr01_42769729	1	0.0000
Chr01_5061855	1	37.9237
Chr01_7241113	1	45.9692
Chr01_7241242	1	45.9692
Chr01_7241235	1	45.9692
Chr01_16667853	1	55.5682
Chr01_12100110	1	64.6408
Chr01_12100213	1	64.6408
Chr01_12100235	1	64.6408
Chr01_51347302	1	75.3269
Chr01_51347327	1	75.3269
Chr01_51347328	1	75.3269
Chr01_39057535	1	98.7837
Chr01_39057596	1	126.2490
Chr01_39057474	1	126.2490
Chr01_39057670	1	130.5906
Chr01_39057649	1	136.7186
Chr01_39057611	1	141.0602
Chr01_39057542	1	141.0602
Chr01_39057613	1	145.4018
Chr01_39057478	1	145.4018
Chr01_39057463	1	145.7668
Chr01_16667945	1	156.4529
Chr01_16667895	1	156.4529
Chr01_16667780	1	156.4529
Chr01_19096761	1	172.7176
Chr01_50073577	1	218.6228
Chr01_50073461	1	219.3582
Chr01_50187545	1	220.0936
Chr01_50146664	1	220.0936
Chr01_50146614	1	220.0936
Chr01_50089253	1	220.8289
Chr01_49935014	1	221.5643
Chr01_49935466	1	221.5643
Chr01_49835865	1	221.9292
Chr01_49872051	1	222.2942
Chr01_49835661	1	222.6592
Chr01_49827999	1	222.6592
Chr01_49613932	1	223.0242
Chr01_49751864	1	223.0242
Chr01_49751816	1	223.0242
Chr01_49614011	1	223.0242

Marker	Linkage group/Chromosome	Genetic distance (cM)
Chr01_49614124	1	223.0242
Chr01_49578497	1	223.0242
Chr01_49578559	1	223.0242
Chr01_49697591	1	223.3891
Chr01_49751572	1	223.7541
Chr01_49570323	1	224.1191
Chr01_49570583	1	224.1191
Chr01_49760004	1	224.1191
Chr01_49421547	1	224.8544
Chr01_49474021	1	224.8544
Chr01_49421615	1	224.8544
Chr01_49421625	1	224.8544
Chr01_49407901	1	225.2194
Chr01_49407964	1	225.2194
Chr01_49407955	1	225.2194
Chr01_49407886	1	225.2194
Chr01_49407903	1	225.2194
Chr01_48445107	1	229.5610
Chr01_6984988	1	267.7124
Chr01_7310409	1	268.4478
Chr01_7172287	1	268.4478
Chr01_7288896	1	268.4478
Chr01_7172201	1	268.4478
Chr01_7633947	1	268.8127
Chr01_7634185	1	268.8127
Chr01_7634098	1	268.8127
Chr01_7172290	1	269.1777
Chr01_7692245	1	269.9130
Chr01_6696033	1	270.6484
Chr01_6696281	1	270.6484
Chr01_6696388	1	270.6484
Chr01_6696365	1	270.6484
Chr01_6904739	1	271.0133
Chr01_6904721	1	271.0133
Chr01_6696371	1	271.3783
Chr01_6463712	1	271.7433
Chr01_6463766	1	271.7433
Chr01_6464042	1	271.7433
Chr01_6463698	1	271.7433
Chr01_6464097	1	271.7433
Chr01_6550303	1	271.7433
Chr01_6018364	1	272.8546
Chr01_5762485	1	273.2195

Marker	Linkage group/Chromosome	Genetic distance (cM)
Chr01_5762498	1	273.2195
Chr01_5762486	1	273.2195
Chr01_5762493	1	273.2195
Chr01_5762497	1	273.2195
Chr01_5762374	1	273.2195
Chr01_5571051	1	273.5845
Chr01_5335920	1	274.6958
Chr01_5381282	1	274.6958
Chr01_5381281	1	274.6958
Chr01_5381243	1	274.6958
Chr01_5335659	1	274.6958
Chr01_5335701	1	274.6958
Chr01_5381283	1	274.6958
Chr01_5381635	1	274.6958
Chr01_4971637	1	274.6958
Chr01_4978730	1	274.6958
Chr01_4980226	1	274.6958
Chr01_4980543	1	274.6958
Chr01_4980560	1	274.6958
Chr01_5003294	1	274.6958
Chr01_4971629	1	274.6958
Chr01_4971719	1	274.6958
Chr01_5332964	1	274.6958
Chr01_5332883	1	274.6958
Chr01_4978868	1	275.0607
Chr01_5332959	1	275.4257
Chr01_5399214	1	275.7906
Chr01_5399181	1	275.7906
Chr01_5399463	1	275.7906
Chr01_5399177	1	275.7906
Chr01_5399202	1	275.7906
Chr01_5399170	1	275.7906
Chr01_5399185	1	275.7906
Chr01_5399146	1	275.7906
Chr01_5399482	1	275.7906
Chr01_5399159	1	275.7906
Chr01_5399147	1	275.7906
Chr01_35896759	1	409.4055
Chr01_35896769	1	409.4055
Chr01_51347326	1	424.9852
Chr01_51347337	1	424.9852
Chr01_51347346	1	424.9852
Chr01_49375521	1	434.5841

Marker	Linkage group/Chromosome	Genetic distance (cM)
Chr01_49375512	1	434.5841
Chr01_49375506	1	434.5841
Chr01_49375484	1	434.5841
Chr01_49375446	1	434.5841
Chr01_49375435	1	434.5841
Chr01_50187716	1	436.4647
Chr01_50187694	1	436.4647
Chr01_39300039	1	443.5363
Chr01_1786889	1	446.2106
Chr01_24267201	1	447.3219
Chr01_24267262	1	447.3219
Chr01_19096806	1	467.8575
Chr01_7811494	1	479.6801
Chr01_14942797	1	489.2791
Chr01_14942783	1	489.2791
Chr01_14942877	1	489.2791
Chr01_14942878	1	489.2791
Chr01_14942887	1	489.2791
Chr01_14942915	1	489.2791
Chr01_14942929	1	489.2791
Chr01_27684594	1	496.3506
Chr01_27684614	1	496.3506
Chr01_36201895	1	501.5695
Chr01_36201894	1	501.5695
Chr01_41948777	1	502.3049
Chr01_5045698	1	507.9742
Chr01_5045706	1	507.9742
Chr01_2270786	1	514.6860
Chr01_47182580	1	517.7668
Chr01_16769433	1	525.3239
Chr01_29187090	1	560.0505
Chr01_29187107	1	560.0505
Chr01_29187133	1	560.0505
Chr01_29187198	1	560.0505
Chr01_29187199	1	560.0505
Chr01_29187211	1	560.0505
Chr01_24577991	1	577.0194
Chr01_42769731	1	586.0920
Chr01_43822756	1	587.5850
Chr01_43822395	1	603.8497
Chr01_49697127	1	637.4431
Chr01_49751789	1	658.2659
Chr01_49751621	1	658.2659

Marker	Linkage group/Chromosome	Genetic distance (cM)
Chr01_49724122	1	658.2659
Chr01_49760375	1	658.6309
Chr01_48339634	1	664.7589
Chr02_47493692	2	0.0000
Chr02_47505908	2	0.0000
Chr02_47566896	2	0.3650
Chr02_49004261	2	8.9221
Chr02_49150877	2	9.6575
Chr02_49087605	2	9.6575
Chr02_49439377	2	10.3928
Chr02_49439157	2	10.3928
Chr02_49439131	2	10.3928
Chr02_49439125	2	10.3928
Chr02_49419053	2	10.3928
Chr02_49419044	2	10.3928
Chr02_49419041	2	10.3928
Chr02_49419004	2	10.3928
Chr02_49418965	2	10.3928
Chr02_49418952	2	10.3928
Chr02_49418943	2	10.3928
Chr02_49418914	2	10.3928
Chr02_49401224	2	10.7578
Chr02_49401074	2	10.7578
Chr02_48767314	2	19.3149
Chr02_48767236	2	19.3149
Chr02_49020237	2	19.6799
Chr02_48705285	2	20.0449
Chr02_48823690	2	27.6019
Chr02_48767503	2	35.1589
Chr02_48545931	2	35.5239
Chr02_48552811	2	35.5239
Chr02_48552814	2	35.5239
Chr02_48640313	2	35.5239
Chr02_48643618	2	35.5239
Chr02_48643786	2	35.5239
Chr02_48645240	2	35.5239
Chr02_43048091	2	49.1546
Chr02_38560323	2	56.2261
Chr02_41751936	2	61.4450
Chr02_41751909	2	61.4450
Chr02_41751898	2	61.4450
Chr02_41751876	2	61.4450
Chr02_41751830	2	61.4450

Marker	Linkage group/Chromosome	Genetic distance (cM)
Chr02_41751825	2	61.4450
Chr02_41751803	2	61.4450
Chr02_41751794	2	61.4450
Chr02_41751774	2	61.4450
Chr02_41751938	2	61.4450
Chr02_35369867	2	67.1143
Chr02_37745825	2	72.7837
Chr02_48949927	2	75.0580
Chr02_10310986	2	83.1100
Chr02_18403464	2	94.9327
Chr02_765970	2	111.9017
Chr02_872596	2	150.0531
Chr02_872663	2	150.0531
Chr02_872686	2	150.0531
Chr02_1135128	2	155.2719
Chr02_1651431	2	162.3435
Chr02_1485115	2	165.0178
Chr02_1931208	2	168.0986
Chr02_3204228	2	184.3633
Chr02_3204321	2	184.3633
Chr02_3204150	2	184.3633
Chr02_3204334	2	184.3633
Chr02_3299297	2	185.8563
Chr02_3288300	2	185.8563
Chr02_3459027	2	186.5916
Chr02_3518930	2	187.7029
Chr02_3958350	2	192.4793
Chr02_16239727	2	203.7272
Chr02_22611601	2	205.2202
Chr02_25620016	2	209.9966
Chr02_25847345	2	210.7320
Chr02_26534346	2	214.6462
Chr02_26534185	2	215.3815
Chr02_26480656	2	219.7231
Chr02_26128905	2	219.7231
Chr02_27099042	2	220.0881
Chr02_26698961	2	220.4531
Chr02_29016823	2	224.7947
Chr02_31959417	2	235.4808
Chr02_32113326	2	239.8223
Chr02_32252177	2	240.1873
Chr02_31873402	2	242.0679
Chr02_34643227	2	255.0813

Marker	Linkage group/Chromosome	Genetic distance (cM)
Chr02_34643222	2	263.1334
Chr02_36996368	2	280.1023
Chr02_35065692	2	288.6595
Chr02_35065730	2	290.1525
Chr02_35071119	2	290.1525
Chr02_34969601	2	290.5174
Chr02_34428762	2	291.6287
Chr02_34518761	2	291.6287
Chr02_35071123	2	293.1217
Chr02_35071362	2	293.1217
Chr02_35130486	2	293.8571
Chr02_35131947	2	294.2220
Chr02_35289581	2	294.9574
Chr02_35369945	2	294.9574
Chr02_35245635	2	295.3223
Chr02_36348041	2	295.6873
Chr02_35423190	2	296.0522
Chr02_36353571	2	296.4172
Chr02_35423272	2	296.7822
Chr02_35437425	2	296.7822
Chr02_35560502	2	296.7822
Chr02_35926043	2	296.7822
Chr02_36209958	2	296.7822
Chr02_36210024	2	296.7822
Chr02_36317988	2	296.7822
Chr02_36348037	2	296.7822
Chr02_36308667	2	297.1471
Chr02_36348048	2	297.5121
Chr02_35437883	2	297.8770
Chr02_36328647	2	298.2420
Chr02_35925992	2	298.6070
Chr02_35926013	2	298.6070
Chr02_36137891	2	299.3423
Chr02_35344261	2	305.9377
Chr02_36750706	2	311.1566
Chr02_36996796	2	313.4308
Chr02_36996756	2	313.4308
Chr02_36996709	2	313.4308
Chr02_36996658	2	313.4308
Chr02_36853901	2	313.4308
Chr02_36853822	2	313.4308
Chr02_37485770	2	314.1662
Chr02_37531627	2	314.5312

Marker	Linkage group/Chromosome	Genetic distance (cM)
Chr02_37504592	2	314.5312
Chr02_37504412	2	314.8961
Chr02_37504523	2	314.8961
Chr02_37504524	2	314.8961
Chr02_37549888	2	314.8961
Chr02_37564453	2	314.8961
Chr02_37564498	2	314.8961
Chr02_37616759	2	314.8961
Chr02_37616866	2	314.8961
Chr02_37616875	2	314.8961
Chr02_37616878	2	314.8961
Chr02_37616898	2	314.8961
Chr02_37819201	2	315.6315
Chr02_37809753	2	315.6315
Chr02_38311276	2	315.9964
Chr02_38422194	2	316.3614
Chr02_38422210	2	316.3614
Chr02_38422327	2	316.3614
Chr02_38422398	2	316.3614
Chr02_38198469	2	316.7263
Chr02_37937595	2	317.0913
Chr02_37937660	2	317.4563
Chr02_37937641	2	317.4563
Chr02_37937590	2	317.4563
Chr02_37874656	2	317.4563
Chr02_38647911	2	317.8212
Chr02_38452857	2	318.1862
Chr02_38564863	2	318.5511
Chr02_37937763	2	322.4654
Chr02_38638752	2	326.3796
Chr02_38512866	2	326.7445
Chr02_38777790	2	327.1095
Chr02_38777589	2	327.1095
Chr02_38987232	2	327.4745
Chr02_38987220	2	327.4745
Chr02_38716362	2	327.4745
Chr02_38716176	2	327.4745
Chr02_39106796	2	327.8394
Chr02_39498546	2	328.5748
Chr02_39410117	2	328.5748
Chr02_39365564	2	328.9397
Chr02_39636012	2	329.6751
Chr02_39784499	2	330.7864

Marker	Linkage group/Chromosome	Genetic distance (cM)
Chr02_39912603	2	330.7864
Chr02_39842055	2	330.7864
Chr02_39842219	2	330.7864
Chr02_40090205	2	331.8977
Chr02_40090218	2	331.8977
Chr02_40090290	2	331.8977
Chr02_40477512	2	332.6330
Chr02_40477223	2	332.6330
Chr02_40364251	2	332.6330
Chr02_40356240	2	332.6330
Chr02_40356238	2	332.6330
Chr02_40356197	2	332.6330
Chr02_40356129	2	332.6330
Chr02_40356128	2	332.6330
Chr02_40731868	2	333.3684
Chr02_40607945	2	333.7333
Chr02_40607973	2	333.7333
Chr02_40608067	2	333.7333
Chr02_40725222	2	334.0983
Chr02_40723162	2	334.4633
Chr02_40723188	2	334.4633
Chr02_40724633	2	334.8282
Chr02_40718639	2	334.8282
Chr02_40693115	2	334.8282
Chr02_41214122	2	335.1932
Chr02_40844547	2	335.5581
Chr02_40828839	2	335.5581
Chr02_40828837	2	335.5581
Chr02_40718552	2	335.9231
Chr02_41084202	2	335.9231
Chr02_41084334	2	335.9231
Chr02_41084337	2	335.9231
Chr02_41106574	2	335.9231
Chr02_41106594	2	335.9231
Chr02_41115157	2	335.9231
Chr02_41115176	2	335.9231
Chr02_41115231	2	335.9231
Chr02_41172368	2	335.9231
Chr02_42072411	2	336.2881
Chr02_41370410	2	339.3689
Chr02_41370426	2	339.3689
Chr02_41656959	2	342.0432
Chr02_42072366	2	342.4081

Marker	Linkage group/Chromosome	Genetic distance (cM)
Chr02_41992594	2	342.4081
Chr02_41982208	2	342.4081
Chr02_41858262	2	342.4081
Chr02_41858233	2	342.4081
Chr02_41857913	2	342.4081
Chr02_41857883	2	342.4081
Chr02_41857849	2	342.4081
Chr02_41812032	2	342.4081
Chr02_41811814	2	342.4081
Chr02_41748916	2	342.4081
Chr02_41748862	2	342.4081
Chr02_41748763	2	342.4081
Chr02_41748716	2	342.4081
Chr02_41734275	2	342.4081
Chr02_41687982	2	342.4081
Chr02_41687965	2	342.4081
Chr02_41687924	2	342.4081
Chr02_41664674	2	342.4081
Chr02_41507256	2	342.4081
Chr02_41506661	2	342.4081
Chr02_41502354	2	342.4081
Chr02_41469102	2	342.4081
Chr02_41469051	2	342.4081
Chr02_41468850	2	342.4081
Chr02_41468848	2	342.4081
Chr02_41468840	2	342.4081
Chr02_41379499	2	342.4081
Chr02_41379400	2	342.4081
Chr02_41365674	2	342.4081
Chr02_42199175	2	342.7731
Chr02_42199058	2	342.7731
Chr02_42160137	2	342.7731
Chr02_42141432	2	342.7731
Chr02_42141416	2	342.7731
Chr02_42111265	2	342.7731
Chr02_42111206	2	342.7731
Chr02_42106827	2	342.7731
Chr02_42106819	2	342.7731
Chr02_42106772	2	342.7731
Chr02_42106750	2	342.7731
Chr02_42106744	2	342.7731
Chr02_42317718	2	343.5084
Chr02_42437618	2	345.3890

Marker	Linkage group/Chromosome	Genetic distance (cM)
Chr02_42313917	2	347.6633
Chr02_42413489	2	348.3987
Chr02_42437630	2	349.1340
Chr02_42609107	2	349.4990
Chr02_42478967	2	349.8639
Chr02_42479044	2	349.8639
Chr02_42479063	2	349.8639
Chr02_42479136	2	349.8639
Chr02_42479142	2	349.8639
Chr02_42479145	2	349.8639
Chr02_42479155	2	349.8639
Chr02_42522538	2	349.8639
Chr02_42603419	2	349.8639
Chr02_42603436	2	349.8639
Chr02_42603672	2	349.8639
Chr02_42603695	2	349.8639
Chr02_42605734	2	349.8639
Chr02_42608152	2	349.8639
Chr02_42611377	2	349.8639
Chr02_42963945	2	350.2289
Chr02_43129030	2	350.9642
Chr02_43129069	2	350.9642
Chr02_42812287	2	351.6996
Chr02_43129392	2	352.0645
Chr02_42963996	2	352.4295
Chr02_42965340	2	352.4295
Chr02_42965628	2	352.4295
Chr02_43008043	2	352.4295
Chr02_43008433	2	352.4295
Chr02_43020459	2	352.4295
Chr02_43020474	2	352.4295
Chr02_43020475	2	352.4295
Chr02_43020477	2	352.4295
Chr02_43020540	2	352.4295
Chr02_43039022	2	352.4295
Chr02_43048038	2	352.4295
Chr02_43050496	2	352.4295
Chr02_43066229	2	352.4295
Chr02_43144420	2	352.4295
Chr02_43714192	2	355.1038
Chr02_42568009	2	368.7345
Chr02_42567735	2	368.7345
Chr02_41768772	2	371.0088

Marker	Linkage group/Chromosome	Genetic distance (cM)
Chr02_41768728	2	371.0088
Chr02_41413481	2	371.0088
Chr02_40802512	2	371.7441
Chr02_39784586	2	381.8807
Chr02_39784517	2	381.8807
Chr02_39785038	2	381.8807
Chr02_40324276	2	388.0088
Chr02_40324291	2	388.0088
Chr02_40324286	2	388.0088
Chr02_10311018	2	393.2277
Chr02_10310968	2	393.2277
Chr02_15346129	2	403.3643
Chr02_15346140	2	403.3643
Chr02_15346211	2	403.3643
Chr02_15346054	2	403.3643
Chr02_15346068	2	403.3643
Chr02_15346197	2	403.3643
Chr02_15346121	2	403.3643
Chr02_15661334	2	409.0336
Chr02_29139352	2	422.6643
Chr02_27123409	2	436.9279
Chr02_33461091	2	449.9413
Chr02_33461093	2	452.2156
Chr02_49473001	2	454.4899
Chr02_38672560	2	457.1642
Chr02_38672521	2	457.1642
Chr02_37551913	2	463.2922
Chr02_37992794	2	472.8912
Chr02_37992778	2	472.8912
Chr02_37992808	2	472.8912
Chr02_37992598	2	485.3021
Chr02_37992662	2	485.3021
Chr02_30494123	2	495.4387
Chr02_30494161	2	495.4387
Chr02_34643107	2	522.0828
Chr02_18256687	2	535.7134
Chr02_26447731	2	574.9518
Chr02_37992782	2	584.5508
Chr02_37992855	2	585.2862
Chr02_45532702	2	588.7802
Chr02_18130197	2	590.2732
Chr02_2223782	2	590.6382
Chr02_48782531	2	593.3125

Marker	Linkage group/Chromosome	Genetic distance (cM)
Chr02_16044524	2	595.5868
Chr02_765964	2	600.9354
Chr02_22458620	2	607.5924
Chr02_49157323	2	618.2785
Chr02_48823751	2	618.6435
Chr02_49004038	2	619.7548
Chr02_49166021	2	620.4901
Chr02_49223414	2	620.8551
Chr02_49223396	2	620.8551
Chr02_49223334	2	620.8551
Chr02_49223278	2	620.8551
Chr02_49223267	2	620.8551
Chr02_49223220	2	620.8551
Chr02_49223217	2	620.8551
Chr02_48823721	2	622.3481
Chr02_48823910	2	622.3481
Chr02_48823820	2	624.6224
Chr02_47556959	2	643.8317
Chr02_47556919	2	643.8317
Chr02_47065872	2	647.3257
Chr02_44759055	2	662.9054
Chr02_44715020	2	662.9054
Chr02_44526027	2	664.0167
Chr02_35846192	2	681.7103
Chr02_34070996	2	686.4867
Chr02_32101230	2	698.3093
Chr02_25847338	2	714.5740
Chr02_23651846	2	718.4883
Chr02_22783578	2	718.8533
Chr02_17974916	2	720.7339
Chr02_15954696	2	723.4082
Chr02_4071337	2	735.2308
Chr02_29261100	2	783.5189
Chr02_4665445	2	797.7825
Chr02_4665728	2	798.8937
Chr02_4659506	2	807.9664
Chr03_1228714	3	0.0000
Chr03_22681883	3	38.1514
Chr03_22730489	3	41.6455
Chr03_22730470	3	41.6455
Chr03_22730465	3	41.6455
Chr03_22730461	3	41.6455
Chr03_22730458	3	41.6455

Marker	Linkage group/Chromosome	Genetic distance (cM)
Chr03_22730430	3	41.6455
Chr03_22730427	3	41.6455
Chr03_22730424	3	41.6455
Chr03_22730399	3	41.6455
Chr03_22730386	3	41.6455
Chr03_22730316	3	41.6455
Chr03_22730311	3	41.6455
Chr03_22502302	3	50.7181
Chr03_22502256	3	50.7181
Chr03_23063296	3	54.2121
Chr03_23653956	3	59.8815
Chr03_31865054	3	75.4612
Chr03_10968105	3	83.5133
Chr03_38749221	3	94.7612
Chr03_52202298	3	104.8978
Chr03_20056360	3	110.5672
Chr03_20056358	3	110.5672
Chr03_20056256	3	110.5672
Chr03_20056394	3	110.5672
Chr03_46989236	3	115.3435
Chr03_23833705	3	118.4244
Chr03_23833723	3	118.4244
Chr03_19460828	3	125.4959
Chr03_16452353	3	134.0531
Chr03_140937	3	149.6328
Chr03_12211975	3	165.2125
Chr03_44999663	3	172.2841
Chr03_39939576	3	174.5584
Chr03_35468421	3	185.2848
Chr03_32329201	3	187.9591
Chr03_16780909	3	197.5580
Chr03_9177319	3	203.2274
Chr03_26094159	3	210.2989
Chr03_26093987	3	210.2989
Chr03_26691340	3	221.5468
Chr03_22020606	3	231.1458
Chr03_16427753	3	237.2739
Chr03_26331668	3	239.9482
Chr03_14969248	3	242.6225
Chr03_14887155	3	242.6225
Chr03_19701297	3	243.3578
Chr03_26331646	3	244.0932
Chr03_15194436	3	244.4582

Marker	Linkage group/Chromosome	Genetic distance (cM)
Chr03_6291829	3	249.6770
Chr03_6348541	3	249.6770
Chr03_6112428	3	250.0420
Chr03_6112490	3	250.0420
Chr03_6112799	3	250.0420
Chr03_9633219	3	255.2609
Chr03_16616870	3	255.9962
Chr03_17264256	3	255.9962
Chr03_18839040	3	255.9962
Chr03_19265038	3	255.9962
Chr03_19460894	3	255.9962
Chr03_19797543	3	255.9962
Chr03_22303899	3	255.9962
Chr03_22368437	3	255.9962
Chr03_22503317	3	255.9962
Chr03_23122375	3	255.9962
Chr03_23132149	3	255.9962
Chr03_23132150	3	255.9962
Chr03_25705964	3	255.9962
Chr03_14741490	3	256.3612
Chr03_14665946	3	256.3612
Chr03_26033564	3	256.7262
Chr03_19043093	3	257.0911
Chr03_28447734	3	257.4561
Chr03_27904401	3	257.8210
Chr03_28230894	3	257.8210
Chr03_31913224	3	261.3151
Chr03_31865107	3	261.3151
Chr03_31585376	3	261.3151
Chr03_33147007	3	262.4264
Chr03_32985006	3	262.7914
Chr03_36780399	3	270.8434
Chr03_37246267	3	273.1177
Chr03_37181875	3	273.4827
Chr03_37393560	3	274.9757
Chr03_38917803	3	277.6500
Chr03_40495420	3	282.8688
Chr03_38337538	3	288.5382
Chr03_16452251	3	332.2682
Chr03_16452359	3	332.2682
Chr03_16452253	3	349.2371
Chr03_52637785	3	358.8361
Chr03_22730448	3	386.3014

Marker	Linkage group/Chromosome	Genetic distance (cM)
Chr03_30221015	3	398.7123
Chr03_29561087	3	399.0773
Chr03_29278472	3	409.2139
Chr03_21716114	3	420.4618
Chr03_22246304	3	421.5731
Chr03_19940645	3	421.5731
Chr03_22503310	3	421.5731
Chr03_21473584	3	421.5731
Chr03_21554809	3	421.5731
Chr03_16113189	3	421.5731
Chr03_22389250	3	421.5731
Chr03_22368469	3	421.5731
Chr03_19940829	3	421.5731
Chr03_22368403	3	421.5731
Chr03_18890989	3	421.5731
Chr03_22742439	3	421.5731
Chr03_18891091	3	421.5731
Chr03_18890946	3	421.5731
Chr03_20648600	3	421.5731
Chr03_18890962	3	421.5731
Chr03_23231768	3	421.5731
Chr03_18891053	3	421.5731
Chr03_20470851	3	421.5731
Chr03_19302598	3	421.5731
Chr03_13998658	3	421.5731
Chr03_19662581	3	421.5731
Chr03_18591301	3	421.5731
Chr03_18699942	3	421.5731
Chr03_18591247	3	421.5731
Chr03_22704084	3	421.5731
Chr03_18891074	3	421.5731
Chr03_26958821	3	421.5731
Chr03_26964552	3	421.5731
Chr03_22704035	3	421.5731
Chr03_15160144	3	421.5731
Chr03_16005796	3	421.5731
Chr03_21310813	3	421.5731
Chr03_16427748	3	421.5731
Chr03_21473583	3	421.5731
Chr03_18891090	3	421.5731
Chr03_19940766	3	421.5731
Chr03_18061775	3	421.9381
Chr03_15160088	3	422.3030

Marker	Linkage group/Chromosome	Genetic distance (cM)
Chr03_11944640	3	422.6680
Chr03_10964387	3	422.6680
Chr03_10940359	3	422.6680
Chr03_11445872	3	423.0330
Chr03_11041115	3	424.5259
Chr03_11944447	3	426.0189
Chr03_10940320	3	426.3839
Chr03_19043097	3	426.7488
Chr03_20556992	3	427.1138
Chr03_20519595	3	430.1946
Chr03_31993097	3	436.3227
Chr03_31910483	3	436.3227
Chr03_32678669	3	437.0580
Chr03_33986137	3	437.7934
Chr03_33931818	3	438.1583
Chr03_34038425	3	438.5233
Chr03_35170919	3	439.2586
Chr03_35826104	3	441.9329
Chr03_36534304	3	444.2072
Chr03_37018284	3	445.7002
Chr03_37034020	3	446.4355
Chr03_37110640	3	447.9285
Chr03_37181800	3	449.0398
Chr03_37601067	3	450.5328
Chr03_39332727	3	454.4470
Chr03_48877722	3	468.7106
Chr04_1289198	4	0.0000
Chr04_97698	4	16.4668
Chr04_43274	4	16.8318
Chr04_53507	4	16.8318
Chr04_53645	4	16.8318
Chr04_432853	4	17.9431
Chr04_432809	4	18.6784
Chr04_432507	4	18.6784
Chr04_386889	4	20.1714
Chr04_249767	4	21.2827
Chr04_432619	4	22.7757
Chr04_140826	4	23.8870
Chr04_539955	4	23.8870
Chr04_152478	4	23.8870
Chr04_152555	4	23.8870
Chr04_152763	4	23.8870
Chr04_145672	4	24.2519

Marker	Linkage group/Chromosome	Genetic distance (cM)
Chr04_266391	4	24.6169
Chr04_157804	4	24.9819
Chr04_157838	4	24.9819
Chr04_121716	4	25.3468
Chr04_1425279	4	25.7118
Chr04_1959008	4	26.0768
Chr04_1958764	4	26.0768
Chr04_1958750	4	26.0768
Chr04_1425329	4	26.4417
Chr04_2516397	4	31.6606
Chr04_2514832	4	32.0256
Chr04_2723122	4	32.7609
Chr04_2654225	4	33.4963
Chr04_2723237	4	35.3768
Chr04_2723265	4	35.3768
Chr04_2723091	4	37.2574
Chr04_2894418	4	38.3687
Chr04_47491880	4	73.3911
Chr04_47502470	4	73.7560
Chr04_47177877	4	77.2501
Chr04_47116362	4	77.2501
Chr04_47116301	4	77.2501
Chr04_46864635	4	79.1307
Chr04_46864606	4	79.1307
Chr04_46843375	4	83.4723
Chr04_46765277	4	94.1584
Chr04_47009251	4	109.0714
Chr04_45079532	4	126.7650
Chr04_44669261	4	135.3222
Chr04_44669247	4	135.3222
Chr04_44669246	4	135.3222
Chr04_44669197	4	135.3222
Chr04_44669182	4	135.3222
Chr04_43896251	4	140.5410
Chr04_43896176	4	140.5410
Chr04_43796121	4	156.1208
Chr04_43796168	4	156.1208
Chr04_43896158	4	171.7005
Chr04_42416150	4	177.8285
Chr04_41856170	4	177.8285
Chr04_10127047	4	183.9566
Chr04_8877603	4	185.0679
Chr04_8087422	4	191.6633

Marker	Linkage group/Chromosome	Genetic distance (cM)
Chr04_3454316	4	220.2399
Chr04_3454438	4	220.6048
Chr04_3454413	4	220.6048
Chr04_3453965	4	220.6048
Chr04_3442719	4	220.6048
Chr04_3442692	4	220.6048
Chr04_3509281	4	222.4854
Chr04_3509296	4	222.4854
Chr04_3442390	4	224.3660
Chr04_3203304	4	225.4773
Chr04_3182918	4	225.4773
Chr04_3182789	4	225.4773
Chr04_3182715	4	225.4773
Chr04_3026509	4	226.2127
Chr04_3026449	4	226.2127
Chr04_3026448	4	226.2127
Chr04_3026428	4	226.2127
Chr04_3026421	4	226.2127
Chr04_3026406	4	226.2127
Chr04_2941475	4	228.8870
Chr04_2925900	4	228.8870
Chr04_2925883	4	228.8870
Chr04_2925654	4	228.8870
Chr04_2925641	4	228.8870
Chr04_2925630	4	228.8870
Chr04_2925568	4	228.8870
Chr04_2826952	4	229.6223
Chr04_2885452	4	229.9873
Chr04_2723069	4	231.0986
Chr04_2738041	4	231.4636
Chr04_2738051	4	231.4636
Chr04_2654234	4	232.1989
Chr04_2577298	4	233.6919
Chr04_2577266	4	233.6919
Chr04_2577249	4	233.6919
Chr04_2577244	4	233.6919
Chr04_2577230	4	233.6919
Chr04_2515139	4	234.8032
Chr04_2577802	4	235.1682
Chr04_2578052	4	235.5331
Chr04_2514808	4	235.8981
Chr04_2514797	4	235.8981
Chr04_2514791	4	235.8981

Marker	Linkage group/Chromosome	Genetic distance (cM)
Chr04_2514790	4	235.8981
Chr04_2514782	4	235.8981
Chr04_2514761	4	235.8981
Chr04_2578113	4	236.2631
Chr04_2577694	4	236.2631
Chr04_2577724	4	236.2631
Chr04_2577768	4	236.2631
Chr04_2577773	4	236.2631
Chr04_2578048	4	236.2631
Chr04_2418542	4	236.6281
Chr04_2418353	4	236.6281
Chr04_266330	4	243.6996
Chr04_266322	4	243.6996
Chr04_1938443	4	246.7804
Chr04_1938413	4	246.7804
Chr04_1286704	4	247.5158
Chr04_540024	4	247.8808
Chr04_266240	4	248.6161
Chr04_432623	4	251.2904
Chr04_387298	4	253.9647
Chr04_266481	4	253.9647
Chr04_266241	4	253.9647
Chr04_386920	4	253.9647
Chr04_249374	4	254.7001
Chr04_541846	4	255.4354
Chr04_541882	4	255.4354
Chr04_1958886	4	255.8004
Chr04_1593371	4	256.1654
Chr04_1910134	4	256.5303
Chr04_1286532	4	256.8953
Chr04_1286722	4	256.8953
Chr04_1360714	4	256.8953
Chr04_1425328	4	259.5696
Chr04_1425426	4	259.5696
Chr04_1425444	4	259.5696
Chr04_432533	4	263.4838
Chr04_1425262	4	266.9778
Chr04_2075287	4	277.1145
Chr04_2075250	4	277.1145
Chr04_2075247	4	277.1145
Chr04_2826985	4	319.3217
Chr04_27975920	4	323.2360
Chr04_27975803	4	323.2360

Marker	Linkage group/Chromosome	Genetic distance (cM)
Chr04_21090729	4	327.1502
Chr04_21090727	4	327.1502
Chr04_9121923	4	339.8736
Chr04_524565	4	350.5597
Chr04_524551	4	350.5597
Chr04_16744997	4	360.6963
Chr04_16744964	4	360.6963
Chr04_16744928	4	360.6963
Chr04_16744951	4	360.6963
Chr04_16745014	4	360.6963
Chr04_16744911	4	360.6963
Chr04_40526473	4	367.7679
Chr04_4660822	4	372.1095
Chr04_4660817	4	372.1095
Chr04_4660818	4	372.1095
Chr04_18204274	4	376.0237
Chr04_18204249	4	376.0237
Chr04_18204242	4	376.0237
Chr04_18204199	4	376.0237
Chr04_46279266	4	378.6980
Chr04_41212171	4	390.6975
Chr04_41212163	4	390.6975
Chr04_27975791	4	394.1915
Chr04_12030347	4	397.2723
Chr04_2515180	4	416.8042
Chr04_2515214	4	416.8042
Chr04_2515179	4	416.8042
Chr04_2514681	4	423.3996
Chr04_33521832	4	435.2222
Chr04_41211358	4	456.0450
Chr04_42684423	4	464.6021
Chr04_42416107	4	468.0962
Chr04_10843094	4	486.5361
Chr04_42674844	4	492.2055
Chr04_42674834	4	492.2055
Chr04_43641907	4	497.8748
Chr04_44087448	4	498.6102
Chr04_44155732	4	498.9751
Chr04_44075861	4	499.7105
Chr04_44096431	4	500.4458
Chr04_44076417	4	500.4458
Chr04_44076401	4	500.4458
Chr04_44076329	4	500.4458

Marker	Linkage group/Chromosome	Genetic distance (cM)
Chr04_44054178	4	500.8108
Chr04_46001791	4	519.2507
Chr04_46009668	4	519.9861
Chr04_47515458	4	538.4260
Chr04_47515570	4	538.7910
Chr04_2885340	4	578.6672
Chr04_2075580	4	583.4436
Chr04_448848	4	586.9376
Chr04_45772739	4	596.5366
Chr04_47010603	4	614.2303
Chr04_47010672	4	640.6316
Chr04_47010688	4	640.6316
Chr04_45275966	4	656.8963
Chr04_24369010	4	686.6359
Chr05_623529	5	0.0000
Chr05_623370	5	0.0000
Chr05_1647342	5	12.4109
Chr05_1647320	5	12.4109
Chr05_1647355	5	12.4109
Chr05_1728256	5	37.7917
Chr05_39106645	5	49.0396
Chr05_10133781	5	52.5337
Chr05_37969231	5	59.1290
Chr05_37969246	5	59.1290
Chr05_34997198	5	65.7244
Chr05_26069059	5	74.7970
Chr05_34831535	5	80.4664
Chr05_2291503	5	83.5472
Chr05_10569404	5	85.0402
Chr05_23311666	5	87.3145
Chr05_39732566	5	91.2287
Chr05_36031122	5	93.9030
Chr05_27262278	5	100.0310
Chr05_27262422	5	100.0310
Chr05_24573008	5	103.1119
Chr05_36614387	5	108.3307
Chr05_37437086	5	109.4420
Chr05_18974461	5	111.7163
Chr05_12510195	5	112.8276
Chr05_38799077	5	114.7082
Chr05_38546075	5	115.0732
Chr05_38546072	5	115.0732
Chr05_38798992	5	115.4381

Marker	Linkage group/Chromosome	Genetic distance (cM)
Chr05_8146477	5	117.7124
Chr05_19520638	5	125.7645
Chr05_19520640	5	125.7645
Chr05_19520641	5	125.7645
Chr05_19520649	5	125.7645
Chr05_19520495	5	126.1295
Chr05_19520556	5	126.1295
Chr05_45532	5	136.8156
Chr05_45488	5	137.1806
Chr05_11445111	5	152.7603
Chr05_11445101	5	169.0250
Chr05_26026223	5	189.0279
Chr05_26026134	5	189.0279
Chr05_22629830	5	194.2468
Chr05_31681432	5	214.2498
Chr05_17545977	5	227.2632
Chr05_17035784	5	229.5374
Chr05_27487392	5	229.9024
Chr05_11498360	5	231.3954
Chr05_26426875	5	232.8884
Chr05_23311649	5	233.9997
Chr05_22794178	5	234.3647
Chr05_24338462	5	235.4760
Chr05_25938962	5	235.4760
Chr05_24267189	5	235.8409
Chr05_34991338	5	239.3350
Chr05_34818123	5	239.7000
Chr05_34831428	5	240.0649
Chr05_35555935	5	241.9455
Chr05_36323410	5	243.8261
Chr05_36312030	5	243.8261
Chr05_36312188	5	244.1911
Chr05_36312213	5	244.1911
Chr05_36312224	5	244.1911
Chr05_36323442	5	244.5560
Chr05_36630855	5	246.8303
Chr05_36645864	5	247.1953
Chr05_36564975	5	247.5603
Chr05_36502025	5	247.9253
Chr05_36501959	5	247.9253
Chr05_36630880	5	248.2902
Chr05_36632022	5	248.2902
Chr05_36649291	5	248.2902

Marker	Linkage group/Chromosome	Genetic distance (cM)
Chr05_36565001	5	248.2902
Chr05_36566255	5	248.2902
Chr05_36630891	5	248.2902
Chr05_36631715	5	248.2902
Chr05_36631825	5	248.2902
Chr05_36631982	5	248.2902
Chr05_36879045	5	249.0256
Chr05_36946384	5	249.3906
Chr05_36946393	5	249.3906
Chr05_37131287	5	249.7555
Chr05_37131317	5	249.7555
Chr05_37131175	5	249.7555
Chr05_37131219	5	249.7555
Chr05_37131220	5	249.7555
Chr05_37131265	5	249.7555
Chr05_37360531	5	250.1205
Chr05_37345519	5	250.1205
Chr05_37360656	5	250.4855
Chr05_37361216	5	250.8505
Chr05_37245561	5	251.2154
Chr05_37245537	5	251.2154
Chr05_37245510	5	251.2154
Chr05_37467219	5	251.5804
Chr05_37497787	5	251.9454
Chr05_37498074	5	251.9454
Chr05_37412467	5	251.9454
Chr05_37414433	5	251.9454
Chr05_37466992	5	251.9454
Chr05_37544430	5	252.3104
Chr05_37644856	5	252.3104
Chr05_37739234	5	252.6753
Chr05_37846930	5	253.0403
Chr05_37885227	5	254.1406
Chr05_37847070	5	254.1406
Chr05_38269773	5	269.9926
Chr05_39634064	5	279.0652
Chr05_39634065	5	279.0652
Chr05_39470354	5	286.6222
Chr05_39470158	5	289.7030
Chr05_39732367	5	290.0680
Chr05_39732319	5	290.0680
Chr05_39732082	5	290.0680
Chr05_39720833	5	290.0680

Marker	Linkage group/Chromosome	Genetic distance (cM)
Chr05_39720832	5	290.0680
Chr05_39720791	5	290.0680
Chr05_39720776	5	290.0680
Chr05_39720775	5	290.0680
Chr05_39470170	5	290.4329
Chr05_39711210	5	290.7979
Chr05_39470196	5	291.1628
Chr05_39470271	5	291.5278
Chr05_39470200	5	291.8928
Chr05_39470206	5	291.8928
Chr05_39470223	5	291.8928
Chr05_39711014	5	291.8928
Chr05_39711038	5	291.8928
Chr05_39744481	5	291.8928
Chr05_39470198	5	292.6281
Chr05_39482004	5	293.3635
Chr05_39479013	5	293.7284
Chr05_39479030	5	293.7284
Chr05_39479033	5	293.7284
Chr05_39479042	5	293.7284
Chr05_39479078	5	293.7284
Chr05_39479093	5	293.7284
Chr05_39508871	5	293.7284
Chr05_39508874	5	293.7284
Chr05_39508880	5	293.7284
Chr05_39470178	5	294.0934
Chr05_39421646	5	294.4583
Chr05_39421656	5	294.4583
Chr05_39325966	5	294.8233
Chr05_39339847	5	294.8233
Chr05_39339886	5	294.8233
Chr05_39339907	5	294.8233
Chr05_39339913	5	294.8233
Chr05_39339919	5	294.8233
Chr05_39339948	5	294.8233
Chr05_39339966	5	294.8233
Chr05_39339968	5	294.8233
Chr05_39340027	5	294.8233
Chr05_39340048	5	294.8233
Chr05_39385959	5	294.8233
Chr05_39408010	5	294.8233
Chr05_39209228	5	295.1883
Chr05_39209459	5	295.1883

Marker	Linkage group/Chromosome	Genetic distance (cM)
Chr05_39209472	5	295.1883
Chr05_39270318	5	295.1883
Chr05_39270392	5	295.1883
Chr05_39270456	5	295.1883
Chr05_39270556	5	295.1883
Chr05_38979323	5	295.5532
Chr05_39003340	5	295.5532
Chr05_39003394	5	295.5532
Chr05_39003412	5	295.5532
Chr05_39003413	5	295.5532
Chr05_39003432	5	295.5532
Chr05_39003565	5	295.5532
Chr05_39003588	5	295.5532
Chr05_39003704	5	295.5532
Chr05_39003726	5	295.5532
Chr05_39003748	5	295.5532
Chr05_39003768	5	295.5532
Chr05_39003785	5	295.5532
Chr05_39003791	5	295.5532
Chr05_39003792	5	295.5532
Chr05_38892618	5	296.6645
Chr05_38892633	5	296.6645
Chr05_38892640	5	296.6645
Chr05_38892645	5	296.6645
Chr05_38892718	5	296.6645
Chr05_38892927	5	296.6645
Chr05_38892969	5	296.6645
Chr05_38893032	5	296.6645
Chr05_38715720	5	297.7758
Chr05_38733624	5	297.7758
Chr05_38733670	5	297.7758
Chr05_38733706	5	297.7758
Chr05_38733721	5	297.7758
Chr05_38759007	5	297.7758
Chr05_38798914	5	297.7758
Chr05_38418724	5	299.2688
Chr05_38536809	5	299.6338
Chr05_38484366	5	299.6338
Chr05_38484326	5	299.6338
Chr05_38484218	5	299.6338
Chr05_38484210	5	299.6338
Chr05_38418759	5	299.9987
Chr05_38418794	5	299.9987

Marker	Linkage group/Chromosome	Genetic distance (cM)
Chr05_38418815	5	299.9987
Chr05_38418820	5	299.9987
Chr05_38418864	5	299.9987
Chr05_38418868	5	299.9987
Chr05_38418879	5	299.9987
Chr05_38418887	5	299.9987
Chr05_38407790	5	300.3637
Chr05_38407821	5	300.3637
Chr05_38407884	5	300.3637
Chr05_38365632	5	300.7286
Chr05_38339651	5	301.4640
Chr05_38339638	5	301.4640
Chr05_38339544	5	301.4640
Chr05_38319535	5	302.5753
Chr05_38205280	5	303.3106
Chr05_38205219	5	303.3106
Chr05_38286634	5	304.0460
Chr05_38286482	5	304.0460
Chr05_38195550	5	304.4109
Chr05_38195379	5	304.4109
Chr05_38269776	5	304.7759
Chr05_38536162	5	318.2666
Chr05_38269801	5	327.8656
Chr05_38269879	5	327.8656
Chr05_38269802	5	327.8656
Chr05_39721734	5	345.5593
Chr05_39230007	5	356.2454
Chr05_38799035	5	369.8760
Chr05_11498463	5	395.2568
Chr05_2291502	5	413.6968
Chr05_39003513	5	420.7683
Chr05_10448374	5	429.8410
Chr05_19520549	5	442.2519
Chr05_19520559	5	442.2519
Chr05_18868614	5	452.9380
Chr05_37969266	5	460.9901
Chr05_37969323	5	460.9901
Chr05_37438997	5	488.4554
Chr05_36565704	5	517.0320
Chr05_38536287	5	530.5082
Chr05_38536272	5	530.5082
Chr05_38269805	5	540.1072
Chr05_39075672	5	544.4489

Marker	Linkage group/Chromosome	Genetic distance (cM)
Chr05_39153144	5	544.4489
Chr05_39905682	5	552.0059
Chr05_39905533	5	552.0059
Chr05_39158693	5	560.5630
Chr05_39209452	5	563.2373
Chr05_39189655	5	563.2373
Chr05_39144053	5	563.6023
Chr05_39144049	5	563.6023
Chr05_39144016	5	563.6023
Chr05_39144011	5	563.6023
Chr05_39144007	5	563.6023
Chr05_39144044	5	563.6023
Chr05_39143993	5	563.6023
Chr05_39209490	5	563.9673
Chr05_39209358	5	563.9673
Chr05_39153545	5	563.9673
Chr05_39153145	5	563.9673
Chr05_39209479	5	563.9673
Chr05_39158003	5	564.3323
Chr05_39158459	5	564.3323
Chr05_39091087	5	564.3323
Chr05_39158315	5	564.3323
Chr05_39091177	5	564.3323
Chr05_39158476	5	564.3323
Chr05_39091159	5	564.3323
Chr05_39091210	5	564.3323
Chr05_39091124	5	564.3323
Chr05_39091111	5	564.3323
Chr05_39091240	5	564.3323
Chr05_39091271	5	564.3323
Chr05_39091264	5	564.3323
Chr05_39023722	5	564.6973
Chr05_39023766	5	564.6973
Chr05_39023770	5	564.6973
Chr05_39023510	5	564.6973
Chr05_39003784	5	564.6973
Chr05_39023799	5	564.6973
Chr05_39023821	5	564.6973
Chr05_39023819	5	564.6973
Chr05_39023792	5	564.6973
Chr05_39023789	5	564.6973
Chr05_39023816	5	564.6973
Chr05_38979212	5	565.0623

Marker	Linkage group/Chromosome	Genetic distance (cM)
Chr05_39003561	5	566.9429
Chr05_39003336	5	566.9429
Chr05_38979060	5	568.8235
Chr05_38364974	5	573.5999
Chr05_38286555	5	573.9648
Chr05_38286536	5	573.9648
Chr05_36594542	5	583.7961
Chr05_36676250	5	584.1611
Chr05_36416745	5	586.0417
Chr05_34991312	5	592.1698
Chr05_34991184	5	592.1698
Chr05_32450826	5	594.8441
Chr05_31202635	5	595.2091
Chr05_31015902	5	595.2091
Chr05_30039829	5	595.9445
Chr05_29504772	5	596.6798
Chr05_29504773	5	596.6798
Chr05_19238819	5	597.7911
Chr05_24402293	5	598.5264
Chr05_25264502	5	599.2618
Chr05_15714615	5	599.9971
Chr05_666529	5	620.8199
Chr05_666604	5	620.8199
Chr05_666589	5	620.8199
Chr05_666526	5	620.8199
Chr05_666726	5	620.8199
Chr05_666553	5	620.8199
Chr05_673021	5	621.1849
Chr05_672790	5	621.1849
Chr05_672843	5	621.1849
Chr05_672793	5	621.1849
Chr06_11147038	6	0.0000
Chr06_13598327	6	36.5379
Chr06_13598278	6	36.9029
Chr06_12357419	6	39.1772
Chr06_11884351	6	39.5422
Chr06_11247217	6	40.2775
Chr06_11457751	6	40.2775
Chr06_10311743	6	40.6425
Chr06_10311723	6	40.6425
Chr06_10311673	6	40.6425
Chr06_10311668	6	40.6425
Chr06_10311666	6	40.6425

Marker	Linkage group/Chromosome	Genetic distance (cM)
Chr06_10311626	6	40.6425
Chr06_10311602	6	40.6425
Chr06_9993489	6	41.0075
Chr06_9598516	6	41.3724
Chr06_4996657	6	42.4837
Chr06_4996666	6	42.4837
Chr06_4996683	6	42.4837
Chr06_5132075	6	42.4837
Chr06_5132108	6	42.4837
Chr06_3881331	6	42.8487
Chr06_2241050	6	43.2137
Chr06_2241090	6	43.2137
Chr06_7444578	6	43.5786
Chr06_7444843	6	43.5786
Chr06_804980	6	43.5786
Chr06_7523831	6	43.5786
Chr06_2068194	6	43.5786
Chr06_7523874	6	43.5786
Chr06_7639390	6	43.5786
Chr06_6960269	6	51.6850
Chr06_6960260	6	51.6850
Chr06_6960153	6	51.6850
Chr06_6960259	6	51.6850
Chr06_7027348	6	52.7963
Chr06_804874	6	52.7963
Chr06_4511150	6	53.1612
Chr06_2372529	6	53.8966
Chr06_7926694	6	54.6319
Chr06_8144805	6	54.6319
Chr06_202121	6	54.6319
Chr06_1504675	6	54.9969
Chr06_1570822	6	56.4899
Chr06_2179083	6	57.6012
Chr06_2179042	6	57.6012
Chr06_5117515	6	59.8755
Chr06_11125594	6	72.2864
Chr06_11147008	6	75.7804
Chr06_11147018	6	77.2734
Chr06_11147037	6	77.2734
Chr06_11147062	6	77.2734
Chr06_11147125	6	77.2734
Chr06_11147130	6	77.2734
Chr06_11147131	6	77.2734

Marker	Linkage group/Chromosome	Genetic distance (cM)
Chr06_11147148	6	77.2734
Chr06_11147170	6	77.2734
Chr06_11147194	6	77.2734
Chr06_11953496	6	79.9477
Chr06_12685271	6	89.5467
Chr06_13708076	6	98.6193
Chr06_13708075	6	100.1123
Chr06_13708079	6	100.1123
Chr06_3620145	6	107.1839
Chr06_11125610	6	120.1973
Chr06_11125508	6	127.2688
Chr06_11125556	6	127.2688
Chr06_11125592	6	127.2688
Chr06_11125601	6	127.2688
Chr06_11144175	6	131.1830
Chr06_9499282	6	132.2943
Chr06_7512117	6	134.9686
Chr06_3620262	6	144.5676
Chr06_3620267	6	144.5676
Chr06_23267080	6	155.2537
Chr06_13708077	6	162.3253
Chr06_5930402	6	168.4533
Chr06_8027852	6	171.5341
Chr06_12244172	6	173.8084
Chr06_16983085	6	176.8893
Chr06_2807093	6	237.7187
Chr06_4981124	6	275.8701
Chr06_4981178	6	276.2350
Chr06_4981184	6	276.2350
Chr06_4981132	6	276.2350
Chr06_4981255	6	276.2350
Chr06_4981144	6	276.2350
Chr06_4981192	6	276.2350
Chr06_4981282	6	276.2350
Chr06_10145393	6	302.6364
Chr06_9449875	6	304.1293
Chr06_804951	6	305.6223
Chr06_5686151	6	305.6223
Chr06_3667779	6	305.9873
Chr06_495353	6	306.3522
Chr06_16733	6	306.7172
Chr06_1374720	6	307.8285
Chr06_14123970	6	311.7427

Marker	Linkage group/Chromosome	Genetic distance (cM)
Chr06_14124318	6	312.1077
Chr06_16976975	6	314.3820
Chr07_94040	7	0.0000
Chr07_41455	7	0.3650
Chr07_3320417	7	54.2316
Chr07_3845608	7	58.5732
Chr07_3842984	7	58.5732
Chr07_3994326	7	58.5732
Chr07_4000907	7	58.5732
Chr07_3998919	7	68.1722
Chr07_3999150	7	68.1722
Chr07_2304021	7	108.0484
Chr07_694745	7	118.1850
Chr07_3845350	7	120.8593
Chr07_3845448	7	120.8593
Chr07_25472421	7	130.4583
Chr07_6299590	7	172.1872
Chr07_4000847	7	179.7442
Chr07_4000845	7	179.7442
Chr07_4000861	7	179.7442
Chr07_4000848	7	179.7442
Chr07_4013666	7	180.1092
Chr07_4013578	7	180.4742
Chr07_4000863	7	181.2095
Chr07_4000940	7	181.2095
Chr07_4001326	7	181.9449
Chr07_4001302	7	181.9449
Chr07_4001229	7	181.9449
Chr07_4000869	7	182.6803
Chr07_4294915	7	192.8169
Chr07_4294817	7	192.8169
Chr07_4294605	7	193.9282
Chr07_4294483	7	193.9282
Chr07_4294579	7	193.9282
Chr07_4048097	7	223.6678
Chr07_35573668	7	225.9421
Chr07_2081481	7	227.4351
Chr07_7961650	7	230.1094
Chr07_17764063	7	241.9320
Chr07_25298541	7	253.1799
Chr07_25298447	7	253.1799
Chr07_25298428	7	253.1799
Chr07_25298427	7	253.1799

Marker	Linkage group/Chromosome	Genetic distance (cM)
Chr07_25298569	7	256.6740
Chr07_15650117	7	269.0849
Chr07_3554390	7	278.1575
Chr07_27944114	7	282.9339
Chr07_39534510	7	292.0065
Chr07_39534529	7	292.0065
Chr07_39064534	7	318.4078
Chr07_39064520	7	318.4078
Chr07_39064496	7	318.4078
Chr07_39064470	7	318.4078
Chr07_39089143	7	325.0032
Chr07_39089142	7	325.0032
Chr07_39089134	7	325.0032
Chr07_39089132	7	325.0032
Chr07_39089106	7	325.0032
Chr07_39089103	7	325.0032
Chr07_39291009	7	326.8838
Chr07_39291024	7	326.8838
Chr07_39291044	7	326.8838
Chr07_39291064	7	326.8838
Chr07_39291073	7	326.8838
Chr07_39291095	7	326.8838
Chr07_39291120	7	326.8838
Chr07_39355768	7	327.2487
Chr07_39504534	7	327.6137
Chr07_39504584	7	327.6137
Chr07_39504669	7	327.6137
Chr07_39508777	7	327.6137
Chr07_39356252	7	327.9787
Chr07_39355881	7	328.3436
Chr07_39265232	7	329.4549
Chr07_39265642	7	329.4549
Chr07_39199104	7	330.1903
Chr07_39199113	7	330.1903
Chr07_39199126	7	330.1903
Chr07_39199162	7	330.1903
Chr07_39199170	7	330.1903
Chr07_39199416	7	330.1903
Chr07_39199499	7	330.1903
Chr07_39199510	7	330.1903
Chr07_39199514	7	330.1903
Chr07_37600627	7	345.7700
Chr07_36653260	7	356.4561

Marker	Linkage group/Chromosome	Genetic distance (cM)
Chr07_36653346	7	356.4561
Chr07_36606955	7	356.8210
Chr07_36606993	7	356.8210
Chr07_36641078	7	356.8210
Chr07_36641094	7	356.8210
Chr07_36641105	7	356.8210
Chr07_36641226	7	356.8210
Chr07_36642579	7	356.8210
Chr07_36642609	7	356.8210
Chr07_1516790	7	414.0260
Chr07_39164101	7	502.5499
Chr08_17449396	8	0.0000
Chr08_17449509	8	2.2743
Chr08_26372132	8	26.6745
Chr08_23994426	8	39.6879
Chr08_62851044	8	59.6909
Chr08_33179134	8	71.5135
Chr08_33179144	8	71.5135
Chr08_33179159	8	71.5135
Chr08_33179166	8	71.5135
Chr08_33179205	8	71.5135
Chr08_33179207	8	71.5135
Chr08_12339438	8	77.1829
Chr08_12488365	8	85.7400
Chr08_12488287	8	85.7400
Chr08_12488250	8	85.7400
Chr08_12488247	8	85.7400
Chr08_12488416	8	86.1050
Chr08_12488285	8	86.4700
Chr08_33393436	8	95.5426
Chr08_33393288	8	95.5426
Chr08_33079494	8	102.6142
Chr08_41965257	8	173.8095
Chr08_41965366	8	197.2662
Chr08_41965288	8	197.2662
Chr08_41965307	8	197.2662
Chr08_41965308	8	197.2662
Chr08_41965323	8	197.2662
Chr08_48440041	8	211.5298
Chr08_17449473	8	243.7715
Chr08_15726857	8	271.2368
Chr08_15726875	8	271.2368
Chr08_6374126	8	292.0596

Marker	Linkage group/Chromosome	Genetic distance (cM)
Chr08_6374057	8	292.0596
Chr08_6374124	8	292.0596
Chr08_6374128	8	292.0596
Chr08_6374020	8	292.0596
Chr08_6374129	8	292.0596
Chr08_41016116	8	309.7532
Chr08_33393417	8	324.6662
Chr08_15233298	8	334.2652
Chr08_15233224	8	334.2652
Chr08_10926144	8	344.4018
Chr08_10926165	8	344.4018
Chr08_10926186	8	344.4018
Chr08_31868383	8	357.5643
Chr08_31868357	8	357.5643
Chr08_39181410	8	360.2386
Chr08_23994451	8	385.1049
Chr08_23994410	8	385.1049
Chr08_23994267	8	385.1049
Chr08_24659982	8	387.7791
Chr08_26763182	8	406.9884
Chr08_41565937	8	422.5681
Chr08_41565987	8	422.5681
Chr08_7547891	8	436.8317
Chr08_7547886	8	436.8317
Chr08_15726692	8	461.2320
Chr08_15726805	8	466.9013
Chr08_15717101	8	488.5716
Chr08_15717086	8	488.5716
Chr08_15717115	8	488.5716
Chr08_15717214	8	488.5716
Chr08_15717136	8	488.5716
Chr08_10851357	8	507.7808
Chr08_10851383	8	507.7808
Chr08_10851294	8	507.7808
Chr08_10851304	8	507.7808
Chr08_10851376	8	507.7808
Chr08_10851329	8	507.7808
Chr08_10851253	8	507.7808
Chr08_10851254	8	507.7808
Chr08_10851287	8	507.7808
Chr08_10851321	8	507.7808
Chr08_33185244	8	517.9175
Chr08_31935096	8	525.4745

Marker	Linkage group/Chromosome	Genetic distance (cM)
Chr08_33204268	8	535.0735
Chr08_33228997	8	539.8499
Chr08_20286627	8	547.9019
Chr08_20286706	8	547.9019
Chr08_24789766	8	556.4590
Chr08_41361569	8	564.5111
Chr08_41361597	8	564.5111
Chr08_26691973	8	570.6392
Chr08_26692021	8	571.3745
Chr08_26803460	8	575.2888
Chr08_26803422	8	575.2888
Chr08_23994404	8	581.4168
Chr08_3640802	8	587.0861
Chr08_3640805	8	587.0861
Chr08_62491552	8	591.8625
Chr08_62491500	8	595.3565
Chr08_24659988	8	600.1329
Chr08_43139628	8	603.6269
Chr08_26315867	8	605.9012
Chr08_50756977	8	608.1755
Chr08_50756978	8	608.1755
Chr08_42015006	8	611.6695
Chr08_23994357	8	614.7503
Chr08_25408068	8	618.2443
Chr08_26688098	8	633.1573
Chr08_26688009	8	633.1573
Chr08_14958690	8	662.8970
Chr08_14958652	8	662.8970
Chr08_14958741	8	662.8970
Chr08_14958818	8	662.8970
Chr09_684728	9	0.0000
Chr09_684802	9	28.5766
Chr09_684733	9	28.5766
Chr09_684797	9	28.5766
Chr09_8156355	9	42.8402
Chr09_8156370	9	43.2052
Chr09_8156412	9	43.5702
Chr09_8156375	9	43.5702
Chr09_8156372	9	43.5702
Chr09_8156423	9	43.5702
Chr09_8156367	9	43.5702
Chr09_8156350	9	43.5702
Chr09_8156356	9	43.5702

Marker	Linkage group/Chromosome	Genetic distance (cM)
Chr09_11076608	9	47.4844
Chr09_14524277	9	51.3986
Chr09_14521032	9	56.1750
Chr09_14521049	9	56.1750
Chr09_14521031	9	56.1750
Chr09_14521023	9	56.1750
Chr09_14521024	9	56.1750
Chr09_14521056	9	56.1750
Chr09_14521021	9	56.1750
Chr09_14521051	9	56.1750
Chr09_3328917	9	58.4493
Chr09_3328944	9	59.5606
Chr09_689769	9	65.2299
Chr09_689743	9	65.2299
Chr09_689784	9	65.2299
Chr09_12340770	9	70.8993
Chr09_8158572	9	74.8135
Chr09_23131077	9	77.8943
Chr09_36875995	9	80.5686
Chr09_30387739	9	82.4492
Chr09_21457946	9	91.3713
Chr09_28853043	9	109.0650
Chr09_28853046	9	109.0650
Chr09_28853053	9	109.0650
Chr09_28853042	9	109.0650
Chr09_1127534	9	128.2742
Chr09_37391824	9	130.1548
Chr09_689732	9	132.8291
Chr09_36803453	9	141.9017
Chr09_37393531	9	175.4951
Chr09_14396957	9	223.7831
Chr09_13851250	9	225.6637
Chr09_13851142	9	225.6637
Chr09_13851130	9	225.6637
Chr09_13851109	9	225.6637
Chr09_17827630	9	250.0640
Chr09_14413295	9	270.0670
Chr09_7153986	9	296.4683
Chr09_7153858	9	296.4683
Chr09_21353248	9	302.5963
Chr09_21353259	9	302.5963
Chr09_15131266	9	332.3359
Chr09_14927587	9	332.7009

Marker	Linkage group/Chromosome	Genetic distance (cM)
Chr09_15147377	9	332.7009
Chr09_13631531	9	333.8122
Chr09_13569085	9	333.8122
Chr09_13348489	9	333.8122
Chr09_13710454	9	333.8122
Chr09_14241149	9	348.7252
Chr09_14021761	9	349.8365
Chr09_14073616	9	350.5718
Chr09_14073270	9	350.5718
Chr09_14073256	9	350.5718
Chr09_14094281	9	351.3072
Chr09_14094304	9	351.3072
Chr09_14094330	9	351.3072
Chr09_14094355	9	351.3072
Chr09_14094358	9	351.3072
Chr09_14094400	9	351.3072
Chr09_14093983	9	352.0425
Chr09_13929663	9	353.1538
Chr09_13927412	9	353.8892
Chr09_14549725	9	355.7697
Chr09_14549671	9	355.7697
Chr09_14549647	9	355.7697
Chr09_14549293	9	355.7697
Chr09_14549239	9	355.7697
Chr09_14302310	9	356.8810
Chr09_14307521	9	357.2460
Chr09_14257096	9	357.6110
Chr09_14413296	9	359.1039
Chr09_14413310	9	359.8393
Chr09_14413346	9	365.9673
Chr09_14413327	9	365.9673
Chr09_14413294	9	365.9673
Chr09_14413293	9	365.9673
Chr09_21431512	9	388.5149
Chr09_21065372	9	388.8799
Chr09_21061231	9	388.8799
Chr09_21061224	9	388.8799
Chr09_21058018	9	388.8799
Chr09_21057981	9	388.8799
Chr09_21057977	9	388.8799
Chr09_21057956	9	388.8799
Chr09_21057945	9	388.8799
Chr09_20865151	9	389.6152

Marker	Linkage group/Chromosome	Genetic distance (cM)
Chr09_21065561	9	390.3506
Chr09_21336320	9	390.7155
Chr09_21393904	9	390.7155
Chr09_21393829	9	392.5961
Chr09_36892380	9	429.1340
Chr09_36943156	9	430.6270
Chr09_37297003	9	433.3013
Chr09_37393466	9	434.7943
Chr09_37393467	9	434.7943
Chr09_37393458	9	434.7943
Chr09_37562630	9	435.1592
Chr09_37559648	9	435.5242
Chr10_44163360	10	0.0000
Chr10_44163306	10	7.0716
Chr10_42980097	10	30.5283
Chr10_42796336	10	33.2026
Chr10_42796346	10	33.2026
Chr10_42796391	10	33.2026
Chr10_42780789	10	34.6956
Chr10_42158874	10	41.2910
Chr10_36936554	10	62.9612
Chr10_16311746	10	65.2355
Chr10_30925792	10	66.3468
Chr10_28672525	10	66.7118
Chr10_25160981	10	67.4471
Chr10_25869761	10	67.4471
Chr10_27497120	10	67.4471
Chr10_9231306	10	68.5584
Chr10_9231678	10	68.5584
Chr10_7225757	10	69.2938
Chr10_674482	10	83.5574
Chr10_223601	10	87.0514
Chr10_382346	10	88.5444
Chr10_2745368	10	96.1014
Chr10_3071850	10	111.6812
Chr10_7976422	10	125.9448
Chr10_7829492	10	138.9581
Chr10_13826228	10	145.0862
Chr10_12412979	10	147.7605
Chr10_12412991	10	150.8413
Chr10_15461213	10	155.1829
Chr10_7829481	10	160.4018
Chr10_17250799	10	163.4826

Marker	Linkage group/Chromosome	Genetic distance (cM)
Chr10_14646320	10	166.1569
Chr10_14646632	10	171.3758
Chr10_246924	10	185.0064
Chr10_246939	10	185.0064
Chr10_246825	10	185.0064
Chr10_246953	10	185.0064
Chr10_246951	10	185.0064
Chr10_44163355	10	196.8291
Chr10_44163292	10	197.9404
Chr10_23090260	10	209.1883
Chr10_382160	10	217.2404
Chr10_381956	10	220.7345
Chr10_223550	10	225.5108
Chr10_39999933	10	232.1062
Chr10_36679047	10	235.6003
Chr10_44163331	10	247.4229
Chr10_44163304	10	270.8797
Chr10_44163289	10	270.8797
Chr10_42497774	10	295.2799
Chr10_43002937	10	312.2489
Chr10_43204200	10	314.9232
Chr10_43204121	10	314.9232
Chr10_16398573	10	349.9455
Chr10_29106685	10	351.0568
Chr10_33258393	10	351.7921
Chr10_33172577	10	352.1571
Chr10_26515975	10	352.8925
Chr10_9588970	10	354.3854
Chr10_9589084	10	354.3854
Chr10_7718371	10	355.4967
Chr10_7229868	10	355.8617
Chr10_7142884	10	355.8617
Chr10_1474862	10	368.2726
Chr10_882694	10	370.5469
Chr10_181788	10	372.4275
Chr10_375925	10	372.7924
Chr10_382208	10	372.7924
Chr10_382078	10	372.7924
Chr10_382286	10	372.7924
Chr10_246950	10	373.5278
Chr10_246870	10	373.5278
Chr10_246875	10	399.9291
Chr10_246824	10	399.9291

Marker	Linkage group/Chromosome	Genetic distance (cM)
Chr10_246866	10	399.9291
Chr10_246883	10	399.9291
Chr10_246826	10	399.9291
Chr10_36144416	10	424.3293
Chr10_36144409	10	424.3293
Chr10_36679010	10	460.8673
Chr10_36679008	10	491.8267
Chr10_15835662	10	507.4064
Chr10_15835762	10	507.4064
Chr10_36679045	10	518.0925
Chr10_31729606	10	529.9151
Chr10_14646635	10	542.3260
Chr10_26164207	10	559.2950
Chr10_10559294	10	567.3470
Chr10_7976376	10	578.5950
Chr10_7976427	10	578.5950
Chr10_7976394	10	578.5950
Chr10_18617025	10	596.2886
Chr10_43557923	10	621.6694
Chr11_32484934	11	0.0000
Chr11_32484898	11	0.0000
Chr11_24074141	11	25.3808
Chr11_18098565	11	34.9797
Chr11_24485850	11	43.0318
Chr11_24485992	11	43.3968
Chr11_24485875	11	43.3968
Chr11_41152165	11	54.6447
Chr11_41152152	11	54.6447
Chr11_41152113	11	54.6447
Chr11_41152092	11	54.6447
Chr11_41152084	11	54.6447
Chr11_41152047	11	54.6447
Chr11_15927065	11	64.2437
Chr11_15927035	11	64.2437
Chr11_37278734	11	69.9130
Chr11_37278735	11	69.9130
Chr11_37278740	11	69.9130
Chr11_37278870	11	69.9130
Chr11_51092570	11	76.5084
Chr11_51092640	11	76.5084
Chr11_23895904	11	80.4226
Chr11_23895948	11	80.4226
Chr11_23895955	11	80.4226

Marker	Linkage group/Chromosome	Genetic distance (cM)
Chr11_23895956	11	80.4226
Chr11_46695588	11	82.3032
Chr11_26063150	11	84.9775
Chr11_26063196	11	84.9775
Chr11_35032972	11	88.0583
Chr11_32203017	11	90.7326
Chr11_35331950	11	94.2267
Chr11_1259272	11	100.8220
Chr11_1259270	11	100.8220
Chr11_1259266	11	100.8220
Chr11_1259276	11	100.8220
Chr11_26440772	11	109.8947
Chr11_26440761	11	121.1426
Chr11_26440787	11	121.1426
Chr11_26440899	11	121.1426
Chr11_22028674	11	144.5993
Chr11_22028703	11	144.5993
Chr11_22028700	11	144.5993
Chr11_22761001	11	152.6514
Chr11_23141174	11	161.2086
Chr11_23348901	11	168.7656
Chr11_23348799	11	168.7656
Chr11_23348929	11	168.7656
Chr11_23348753	11	168.7656
Chr11_21542673	11	177.8382
Chr11_2302038	11	186.3954
Chr11_497609	11	202.6601
Chr11_4751895	11	213.3462
Chr11_37865248	11	221.9034
Chr11_37865291	11	221.9034
Chr11_37865294	11	221.9034
Chr11_37865295	11	221.9034
Chr11_37865328	11	221.9034
Chr11_37865337	11	221.9034
Chr11_473239	11	246.3036
Chr11_473334	11	246.3036
Chr11_482505	11	246.3036
Chr11_1285764	11	249.7976
Chr11_982792	11	263.4283
Chr11_2313784	11	285.9759
Chr11_2382238	11	289.8901
Chr11_2419651	11	290.2550
Chr11_2448093	11	290.6200

Marker	Linkage group/Chromosome	Genetic distance (cM)
Chr11_2435242	11	290.9850
Chr11_3260440	11	296.2038
Chr11_3238161	11	296.2038
Chr11_4103315	11	301.8732
Chr11_42487761	11	350.1612
Chr11_42487756	11	350.1612
Chr11_42487799	11	350.1612
Chr11_42487785	11	383.7546
Chr11_42487757	11	383.7546
Chr11_42487728	11	383.7546
Chr11_33686091	11	407.2113
Chr11_33685994	11	407.2113
Chr11_37865243	11	432.5921
Chr11_48352437	11	452.5951
Chr11_18145008	11	456.9366
Chr11_51718031	11	472.5164
Chr11_32484897	11	491.7256
Chr11_26622766	11	509.4193
Chr11_1095432	11	527.1129
Chr11_140358	11	535.1650
Chr11_473259	11	546.4129
Chr11_473241	11	546.4129
Chr11_468484	11	547.5242
Chr11_458691	11	548.6354
Chr11_465161	11	549.7467
Chr11_465399	11	549.7467
Chr11_465411	11	549.7467
Chr11_465417	11	549.7467
Chr11_273955	11	550.4821
Chr11_182056	11	550.4821
Chr11_273943	11	550.4821
Chr11_273877	11	550.4821
Chr11_273776	11	550.4821
Chr11_248569	11	550.4821
Chr11_238590	11	550.4821
Chr11_238554	11	550.4821
Chr11_233454	11	550.4821
Chr11_238525	11	550.4821
Chr11_238519	11	550.4821
Chr11_233555	11	550.4821
Chr11_238186	11	550.4821
Chr11_238320	11	550.4821
Chr11_238240	11	550.4821

Marker	Linkage group/Chromosome	Genetic distance (cM)
Chr11_238287	11	550.4821
Chr11_182185	11	550.4821
Chr11_182141	11	550.4821
Chr11_182211	11	550.4821
Chr11_182074	11	550.4821
Chr11_181992	11	550.4821
Chr11_171523	11	550.4821
Chr11_147523	11	550.8470
Chr11_147591	11	550.8470
Chr11_147530	11	550.8470
Chr11_147443	11	550.8470
Chr11_147422	11	550.8470
Chr11_50534	11	550.8470
Chr11_78636	11	550.8470
Chr11_50525	11	550.8470
Chr11_32586	11	550.8470
Chr11_32488	11	550.8470
Chr11_147600	11	550.8470
Chr11_17856	11	553.5214
Chr11_147405	11	556.1957
Chr11_238479	11	556.5607
Chr11_274004	11	556.5607
Chr11_465463	11	557.2960
Chr11_312041	11	557.6610
Chr11_986764	11	571.2917
Chr11_1276847	11	573.1723
Chr11_1276594	11	587.4359
Chr11_1276342	11	587.4359
Chr11_1276317	11	587.4359
Chr11_1276300	11	587.4359
Chr11_2313817	11	611.8362
Chr11_2313854	11	611.8362
Chr11_2313872	11	611.8362
Chr11_2313880	11	611.8362
Chr11_2302047	11	614.9170
Chr11_2302076	11	614.9170
Chr11_2302126	11	614.9170
Chr11_2301919	11	614.9170
Chr11_4256026	11	627.9304
Chr11_5002346	11	662.9527
