# Supplementary material for: Quantitative Trait Locus Mapping of Marsh Spot Disease Resistance in Cranberry Common Bean (Phaseolus vulgaris L.)
Source: Int J Mol Sci. 2022 Jul 11;23(14):7639. doi: 10.3390/ijms23147639 (PMC9324509; doi:10.3390/ijms23147639)
Supplement: Supplementary file 1 [file ijms-23-07639-s001.zip › ijms-1758465-supplementary/Table_S4.pdf]

Table S4. R^2 of 16 quantitative trait loci (QTL) across 5 years and two soil types (sandy and heavy clay).

Dataset/Q	*QTL.1.1*	*QTL.2.1*	*QTL.2.2*	*QTL.2.3*	*QTL.2.4*	*QTL.2.5*	*QTL.2.6*	*QTL.3.1*	*QTL.3.2*	*QTL.5.1*	*QTL.5.2*	*QTL.5.3*	*QTL.5.4*	*QTL.6.1*	*QTL.6.2*
H2015	4.58	6.52 *	7.88 ***	10.48 **	10.58 ***	9.59 **	9.15 **	27.3 ***	24.99 ***	4.71 *	5.74 *	10.32 ***	6.65 *	1.71	2.61
H2016	2.81	7.3 *	9 ***	9.87 **	9.88 ***	9.49 **	11.47 ***	18.7 ***	17.27 ***	0.73	11.74 ***	7.54 **	6.99 *	8.21 **	2.02
H2017	5.37	4.13	12.04 ***	11.83 ***	11.64 ***	10.21 ***	10.26 **	15.83 **	14.41 ***	2.81	10.13 **	6.6 **	10.89 **	8.16 **	6.06 *
H2018	3.29	8.83 **	8.41 ***	10.8 **	10.28 ***	11.32 ***	9.72 **	13.49 *	11.76 ***	0.81	5.78 *	4.79 **	13.07 ***	1.92	2.42
H2019	3.86	2.96	8.12 ***	11.81 ***	12.53 ***	13.21 ***	12.81 ***	17.26 **	14.15 ***	6.25 *	7.55 *	0.77	12.45 ***	5.53 *	9.14 **
S2015	3.16	1.49	6.39 **	8.24 **	8.22 **	6.04 *	13.42 ***	43.92 ***	42 ***	10.3 ***	7.92 *	6.86 **	12.2 ***	6.72 **	6.34 *
S2016	3.93	7.27 *	11.18 ***	12.49 ***	11.27 ***	8.51 **	13.92 ***	26.14 ***	19.22 ***	1.11	10.22 **	9.82 ***	7.43 *	2.16	2.55
S2017	5.06	10.81 **	4.97 **	7 *	4.61 *	5.59 *	8.74 **	16.82 **	15.54 ***	1.59	7.1 *	21.69 ***	9.94 **	4.42 *	1.69
S2018	4.29	4.57	1.55	3.8	4.52 *	2.09	6.71 *	27.48 ***	22.03 ***	3.19	7.58 *	10.92 ***	10.55 **	9.27 **	6.01 *
S2019	4.84	3.83	4.67 *	5.28	5.02 *	5.75 *	11.04 **	5.28	1.5	0.93	7.96 *	11.11 ***	5.39	4.07	4.01
T2015	3.94	4.52	8.86 ***	11.59 ***	11.61 ***	9.69 **	12.25 ***	39.01 ***	38.14 ***	8.31 **	7.92 *	10.58 ***	10.74 **	4.25	4.75
T2016	3.95	8.57 **	12.42 ***	13.79 ***	12.98 ***	10.64 ***	15.47 ***	25.15 ***	22.04 ***	0.96	12.63 ***	10.37 ***	8.54 **	4.87 *	2.74
T2017	5.9 *	9.39 **	10.38 ***	10.71 **	9.8 ***	9.58 **	11.98 ***	21.08 ***	19.67 ***	2.36	10.85 **	17.9 ***	13.67 ***	7.73 **	3.49
T2018	5.14	9.37 **	7.06 **	10.48 **	10.69 ***	9.22 **	10.21 **	23.34 ***	20.45 ***	2.03	8.12 **	9.91 ***	16.29 ***	6.05 *	5.28
T2019	5.98 *	4.86	10.08 ***	12.69 ***	12.97 ***	14.33 ***	16.31 ***	16.58 **	11.58 ***	2.77	10.19 **	5.03 **	13.7 ***	7.44 **	10.06 **
H-5 yrs	4.95	7.4 *	12.77 ***	15.81 ***	15.94 ***	15.42 ***	15.09 ***	23.47 ***	22.01 ***	3.61	9.99 **	7.02 **	13.83 ***	5.4 *	5.49
S-5 yrs	5.32	7.36 *	8.53 ***	10.4 **	10.03 ***	8.16 **	16.27 ***	31.1 ***	27.29 ***	3.45	12.31 ***	18.51 ***	14.48 ***	7.64 **	5.11
Overall	5.87 *	8.44 **	12.24 ***	14.86 ***	14.94 ***	13.53 ***	16.52 ***	30.1 ***	27.73 ***	3.86	12.44 ***	13.19 ***	16.02 ***	7.08 **	5.54
Mean R^2	5.92	8.30	9.12	11.43	10.42	10.02	12.30	24.52	21.78	7.39	9.23	10.72	11.61	6.81	7.52

Note: *, p<0.05; **, p<0.01; ***,p<0.001, mean R^2 values were calculated from tose datasets which were significant correlation with QTL.

QTL.9.1	Mean R^2
0.27	8.42
2.12	8.20
0.48	8.30
4.28	7.18
0.07	8.15
1.6	10.91
1.79	8.93
9.67 **	7.96
6.51 *	7.72
3.77	5.00
0.69	11.00
2.29	10.09
4.35	9.94
7.27 *	9.48
0.66	9.14
1.03	10.56
4.23	11.22
2.35	12.07
7.82	10.54
