# Supplementary material for: Quantitative Trait Locus Mapping of Marsh Spot Disease Resistance in Cranberry Common Bean (Phaseolus vulgaris L.)
Source: Int J Mol Sci. 2022 Jul 11;23(14):7639. doi: 10.3390/ijms23147639 (PMC9324509; doi:10.3390/ijms23147639)
Supplement: Supplementary file 1 [file ijms-23-07639-s001.zip › ijms-1758465-supplementary/Table_S5.pdf]

Table S5. Relative contribution (%) of each quantitative trait loci (QTL) to the marsh spot resistance index (MSRI) of the 138 recombinant inbred lines (RILs) across 5 years and two soil types (sandy and heavy clay).

Dataset/C	*QTL.1.1*	*QTL.2.1*	*QTL.2.2*	*QTL.2.3*	*QTL.2.4*	*QTL.2.5*	*QTL.2.6*	*QTL.3.1*	*QTL.3.2*	*QTL.5.1*	*QTL.5.2*	*QTL.5.3*	*QTL.5.4*	*QTL.6.1*	*QTL.6.2*	*QTL.9.1*
H2015	6.71	10.72	4.77	5.58	3.27	5.69	8.96	11.07	10.19	1.65	4.46	15.31	8.01	2.21	1.24	0.16
H2016	1.18	11.61	5.27	4.75	2.57	6.07	15.83	8.73	13.62	0.90	7.86	6.31	6.71	3.43	1.83	3.34
H2017	6.11	5.86	9.02	6.29	3.08	5.48	5.25	10.52	4.89	1.19	9.60	6.04	10.63	9.68	6.22	0.15
H2018	5.16	17.99	4.49	5.52	4.67	4.45	7.47	8.33	7.14	0.93	3.94	4.24	19.16	1.96	2.61	1.94
H2019	7.35	6.39	4.08	5.31	5.79	8.22	7.72	8.68	8.31	4.94	5.86	0.66	15.10	5.43	6.03	0.12
S2015	1.28	3.63	3.82	3.91	2.30	7.11	9.65	6.72	10.66	2.89	7.26	8.64	17.12	8.67	5.28	1.06
S2016	2.17	8.80	6.05	6.12	2.97	7.88	15.99	7.65	12.58	0.93	8.23	9.05	5.68	1.59	2.91	1.41
S2017	6.70	8.84	2.34	4.05	2.68	5.15	5.92	2.84	5.86	1.13	3.95	26.14	13.50	6.56	2.62	1.72
S2018	5.38	10.20	1.72	2.13	1.58	4.58	5.62	7.17	4.20	1.20	8.07	18.08	13.67	9.87	5.67	0.86
S2019	10.27	7.92	3.56	4.50	2.61	7.98	2.78	3.62	2.12	5.69	6.49	16.82	9.83	4.86	5.97	5.01
T2015	3.89	7.29	4.56	5.03	2.93	6.20	9.49	9.23	10.65	2.14	5.96	12.54	12.21	4.70	2.83	0.33
T2016	1.52	9.89	5.89	5.61	2.94	7.34	16.11	8.61	13.18	0.64	8.11	7.60	6.14	2.05	2.20	2.17
T2017	7.04	8.15	4.73	5.07	2.66	5.23	6.13	5.79	5.92	0.81	6.64	16.75	13.18	8.22	3.09	0.60
T2018	5.88	16.01	2.80	4.09	2.83	4.61	6.76	8.07	5.75	1.10	5.35	9.30	17.48	4.60	3.75	1.61
T2019	9.97	8.07	4.46	5.53	3.46	9.65	5.83	7.51	5.09	1.23	6.94	2.75	15.64	6.13	7.14	0.60
H-5 yrs	5.58	10.91	5.60	6.01	3.91	6.28	9.02	10.11	9.08	1.51	6.00	4.60	13.08	4.31	3.67	0.33
S-5 yrs	5.03	8.85	3.18	4.13	2.20	7.09	8.20	5.72	6.85	0.87	7.57	16.63	13.30	5.95	3.54	0.90
Overall	5.55	10.33	4.42	5.14	2.67	6.56	8.93	8.14	8.27	1.04	6.79	9.45	13.48	5.01	3.59	0.63

H: heavy clay soil; S: sandy soil; T: means of years over two soil types; H-5 yrs: means of heavy clay soil over five years; S-5 yrs: means of sandy soil over five years; Overall: means over five years and two soil types.
