# Supplementary material for: Quantitative Trait Locus Mapping of Marsh Spot Disease Resistance in Cranberry Common Bean (Phaseolus vulgaris L.)
Source: Int J Mol Sci. 2022 Jul 11;23(14):7639. doi: 10.3390/ijms23147639 (PMC9324509; doi:10.3390/ijms23147639)
Supplement: Supplementary file 1 [file ijms-23-07639-s001.zip › ijms-1758465-supplementary/Table_S6.pdf]

Table S6. Candidate genes across whole reference genome.

ID	Chr	Pos	Gene families
Phvul.001G088900	1	14088551	YELLOW STRIPE like 6
Phvul.001G105400	1	26290093	calcium exchanger 7
Phvul.001G105400	1	26290093	calcium exchanger 7
Phvul.001G142750	1	38584393	Cation efflux family protein
Phvul.001G177500	1	43453927	NRAMP metal ion transporter family protein
Phvul.001G179100	1	43666739	Heavy metal transport/detoxification superfamily protein
Phvul.001G179100	1	43666739	Heavy metal transport/detoxification superfamily protein
Phvul.001G205300	1	46324641	Heavy metal transport/detoxification superfamily protein
Phvul.001G247400	1	49894723	Heavy metal transport/detoxification superfamily protein
Phvul.001G250300	1	50100939	Heavy metal transport/detoxification superfamily protein
Phvul.001G250300	1	50100939	Heavy metal transport/detoxification superfamily protein
Phvul.001G250300	1	50100939	Heavy metal transport/detoxification superfamily protein
Phvul.002G033800	2	3382174	Heavy metal transport/detoxification superfamily protein
Phvul.002G033800	2	3382174	Heavy metal transport/detoxification superfamily protein
Phvul.002G099700	2	19275657	zinc transporter 10 precursor
Phvul.002G100001	2	19321798	zinc transporter 10 precursor
Phvul.002G113500	2	24370429	Vacuolar iron transporter (VIT) family protein
Phvul.002G176300	2	33458683	Cation efflux family protein
Phvul.002G184200	2	34468080	ZIP metal ion transporter family
Phvul.002G205000	2	37162538	Vacuolar iron transporter (VIT) family protein
Phvul.002G205100	2	37176246	Vacuolar iron transporter (VIT) family protein
Phvul.002G205200	2	37188537	Vacuolar iron transporter (VIT) family protein
Phvul.002G205300	2	37195863	Vacuolar iron transporter (VIT) family protein
Phvul.002G208500	2	37516072	Heavy metal transport/detoxification superfamily protein
Phvul.002G262500	2	43427768	Heavy metal transport/detoxification superfamily protein
Phvul.002G285600	2	45486348	Heavy metal transport/detoxification superfamily protein
Phvul.002G292900	2	46189860	Heavy metal transport/detoxification superfamily protein
Phvul.002G314600	2	48136994	Heavy metal transport/detoxification superfamily protein
Phvul.002G322800	2	48774556	vacuolar iron transporter 1
Phvul.002G322900	2	48779470	vacuolar iron transporter 1
Phvul.002G323700	2	48857307	vacuolar iron transporter 1
Phvul.003G006400	3	760614	YELLOW STRIPE like 7
Phvul.003G006500	3	765645	YELLOW STRIPE like 7
Phvul.003G006500	3	765645	YELLOW STRIPE like 7
Phvul.003G021300	3	2036314	Cation efflux family protein
Phvul.003G022400	3	2135327	Heavy metal transport/detoxification superfamily protein
Phvul.003G086300	3	16877488	Heavy metal transport/detoxification superfamily protein
Phvul.003G092500	3	21113140	Heavy metal transport/detoxification superfamily protein
Phvul.003G099700	3	25708027	Heavy metal transport/detoxification superfamily protein
Phvul.003G104900	3	23350962	Heavy metal transport/detoxification superfamily protein
Phvul.003G108900	3	27536901	Heavy metal transport/detoxification superfamily protein
Phvul.003G108900	3	27536901	Heavy metal transport/detoxification superfamily protein

ID	Chr	Pos	Gene families
Phvul.003G108900	3	27536901	Heavy metal transport/detoxification superfamily protein
Phvul.003G219900	3	44817176	Heavy metal transport/detoxification superfamily protein
Phvul.003G262400	3	50155639	zinc transporter 10 precursor
Phvul.003G262500	3	50167952	zinc transporter 10 precursor
Phvul.004G001200	4	36383	Heavy metal transport/detoxification superfamily protein
Phvul.004G019800	4	2331722	Heavy metal transport/detoxification superfamily protein
Phvul.004G049800	4	6070640	calcium exchanger 7
Phvul.004G052700	4	7006062	Heavy metal transport/detoxification superfamily protein
Phvul.004G077300	4	13645167	Heavy metal transport/detoxification superfamily protein
Phvul.004G096500	4	15848521	Vacuolar iron transporter (VIT) family protein
Phvul.004G106900	4	35338479	Heavy metal transport/detoxification superfamily protein
Phvul.004G116300	4	40009283	Heavy metal transport/detoxification superfamily protein
Phvul.004G138900	4	43873548	YELLOW STRIPE like 7
Phvul.004G161300	4	46564258	Heavy metal transport/detoxification superfamily protein
Phvul.005G048900	5	5682746	zinc transporter 1 precursor
Phvul.005G049300	5	5742223	Cation efflux family protein
Phvul.005G095400	5	29797811	Heavy metal transport/detoxification superfamily protein
Phvul.005G131500	5	37041480	Heavy metal transport/detoxification superfamily protein
Phvul.005G182000	5	40667944	NRAMP metal ion transporter 6
Phvul.005G182000	5	40667944	NRAMP metal ion transporter 6
Phvul.005G184600	5	40802614	cation exchanger 2
Phvul.006G001000	6	321846	zinc transporter 1 precursor
Phvul.006G015061	6	2386258	YELLOW STRIPE like 1
Phvul.006G060800	6	16929318	cation exchanger 5
Phvul.006G060800	6	16929318	cation exchanger 5
Phvul.006G067900	6	17933066	cation exchanger 1
Phvul.006G070200	6	18248775	ZIP metal ion transporter family
Phvul.006G080700	6	19279972	Cation efflux family protein
Phvul.006G080700	6	19279972	Cation efflux family protein
Phvul.006G083800	6	19564418	YELLOW STRIPE like 7
Phvul.006G093300	6	20450587	cation exchanger 11
Phvul.006G139900	6	24687127	Heavy metal transport/detoxification superfamily protein
Phvul.006G139900	6	24687127	Heavy metal transport/detoxification superfamily protein
Phvul.006G139900	6	24687127	Heavy metal transport/detoxification superfamily protein
Phvul.006G153400	6	25863506	Heavy metal transport/detoxification superfamily protein
Phvul.007G079100	7	7671485	Vacuolar iron transporter (VIT) family protein
Phvul.008G015500	8	1278190	Cation efflux family protein
Phvul.008G015500	8	1278190	Cation efflux family protein
Phvul.008G015500	8	1278190	Cation efflux family protein
Phvul.008G015500	8	1278190	Cation efflux family protein
Phvul.008G015500	8	1278190	Cation efflux family protein
Phvul.008G058500	8	5220363	Heavy metal transport/detoxification superfamily protein
Phvul.008G070000	8	6339674	vacuolar iron transporter 1

ID	Chr	Pos	Gene families
Phvul.008G079500	8	7719539	ZIP metal ion transporter family
Phvul.008G104500	8	11470299	cation exchanger 1
Phvul.008G157800	8	28064702	YELLOW STRIPE like 3
Phvul.008G157800	8	28064702	YELLOW STRIPE like 3
Phvul.008G157800	8	28064702	YELLOW STRIPE like 3
Phvul.008G187600	8	52505573	Vacuolar iron transporter (VIT) family protein
Phvul.008G187600	8	52505573	Vacuolar iron transporter (VIT) family protein
Phvul.008G187600	8	52505573	Vacuolar iron transporter (VIT) family protein
Phvul.008G187600	8	52505573	Vacuolar iron transporter (VIT) family protein
Phvul.008G187600	8	52505573	Vacuolar iron transporter (VIT) family protein
Phvul.008G187600	8	52505573	Vacuolar iron transporter (VIT) family protein
Phvul.008G187600	8	52505573	Vacuolar iron transporter (VIT) family protein
Phvul.008G189300	8	52746631	Heavy metal transport/detoxification superfamily protein
Phvul.008G189400	8	52754968	Heavy metal transport/detoxification superfamily protein
Phvul.008G216200	8	56526044	Heavy metal transport/detoxification superfamily protein
Phvul.008G219600	8	56886320	Heavy metal transport/detoxification superfamily protein
Phvul.008G244200	8	59266794	Cation efflux family protein
Phvul.008G254900	8	60275655	Cation efflux family protein
Phvul.008G259200	8	60615253	ZIP metal ion transporter family
Phvul.008G261500	8	60843461	cation exchanger 1
Phvul.009G027600	9	6422756	Heavy metal transport/detoxification superfamily protein
Phvul.009G038800	9	8217549	Heavy metal transport/detoxification superfamily protein
Phvul.009G038800	9	8217549	Heavy metal transport/detoxification superfamily protein
Phvul.009G038800	9	8217549	Heavy metal transport/detoxification superfamily protein
Phvul.009G038800	9	8217549	Heavy metal transport/detoxification superfamily protein
Phvul.009G040800	9	8411105	vacuolar iron transporter (VIT) family protein
Phvul.009G040800	9	8411105	vacuolar iron transporter (VIT) family protein
Phvul.009G040800	9	8411105	vacuolar iron transporter (VIT) family protein
Phvul.009G048800	9	9593088	YELLOW STRIPE like 3
Phvul.009G048800	9	9593088	YELLOW STRIPE like 3
Phvul.009G069700	9	12141306	NRAMP metal ion transporter 2
Phvul.009G127900	9	19429094	NRAMP metal ion transporter 6
Phvul.009G127900	9	19429094	NRAMP metal ion transporter 6
Phvul.009G137100	9	20653368	manganese tracking factor for mitochondrial SOD2
Phvul.009G164900	9	24527982	Heavy metal transport/detoxification superfamily protein
Phvul.009G247600	9	36846305	Heavy metal transport/detoxification superfamily protein
Phvul.010G001500	10	412065	cation exchanger 3
Phvul.010G001500	10	412065	cation exchanger 3
Phvul.010G021600	10	3076479	Vacuolar iron transporter (VIT) family protein
Phvul.010G021700	10	3083937	vacuolar iron transporter (VIT) family protein
Phvul.010G034132	10	4957830	Heavy metal transport/detoxification superfamily protein
Phvul.010G059200	10	9326694	ZIP metal ion transporter family
Phvul.010G103800	10	37309047	Heavy metal transport/detoxification superfamily protein

ID	Chr	Pos	Gene families
Phvul.010G103800	10	37309047	Heavy metal transport/detoxification superfamily protein
Phvul.010G110500	10	38414889	NRAMP metal ion transporter 2
Phvul.010G119900	10	40027163	metal tolerance protein A2
Phvul.010G119900	10	40027163	metal tolerance protein A2
Phvul.010G119900	10	40027163	metal tolerance protein A2
Phvul.010G119900	10	40027163	metal tolerance protein A2
Phvul.010G128500	10	40925634	cation calcium exchanger 4
Phvul.010G160800	10	43922651	NRAMP metal ion transporter 6
Phvul.010G160800	10	43922651	NRAMP metal ion transporter 6
Phvul.010G160800	10	43922651	NRAMP metal ion transporter 6
Phvul.011G058500	11	5228604	ZIP metal ion transporter family
Phvul.011G061200	11	5460629	metal tolerance protein B1
Phvul.011G061200	11	5460629	metal tolerance protein B1
Phvul.011G068500	11	6085721	Heavy metal transport/detoxification superfamily protein
Phvul.011G084200	11	7945552	Heavy metal transport/detoxification superfamily protein
Phvul.011G088100	11	8454876	vacuolar protein sorting 41
Phvul.011G178800	11	48990728	Cation efflux family protein
Phvul.011G178800	11	48990728	Cation efflux family protein
Phvul.L001741	scaffold_142	17190	Heavy metal transport/detoxification superfamily protein
Phvul.L002137	scaffold_13	221624	Heavy metal transport/detoxification superfamily protein
Phvul.L005301	scaffold_14	415497	YELLOW STRIPE like 1
Phvul.L005301	scaffold_14	415497	YELLOW STRIPE like 1
Phvul.L007443	scaffold_15	499839	zinc transporter 11 precursor
Phvul.L007543	scaffold_15	504259	zinc transporter 11 precursor
Phvul.L011443	scaffold_15	796287	ZIP metal ion transporter family
