# Supplementary figures and images for: Quantitative Trait Locus Mapping of Marsh Spot Disease Resistance in Cranberry Common Bean (Phaseolus vulgaris L.)
Source: Int J Mol Sci. 2022 Jul 11;23(14):7639. doi: 10.3390/ijms23147639 (PMC9324509; doi:10.3390/ijms23147639)

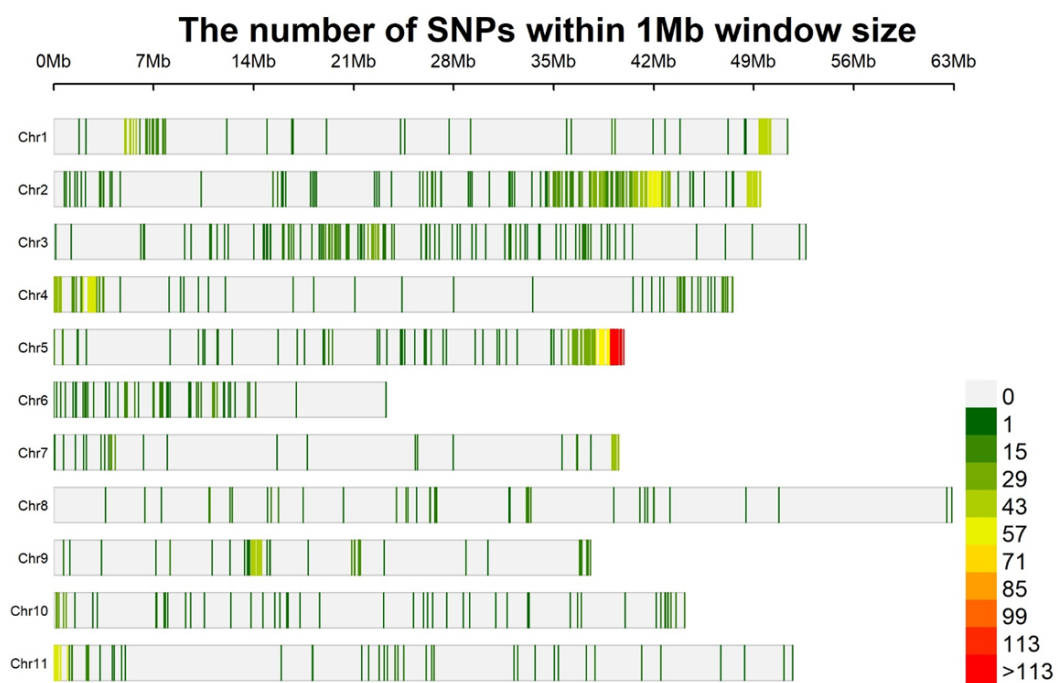

**Figure S1.** Distribution of single nucleotide polymorphisms (SNPs) on 11 common bean chromosomes.

Supplement: Supplementary file 1 [file ijms-23-07639-s001.zip › ijms-1758465-supplementary/Figure_S1.pdf]
